# Two-Phase Analysis in Consensus Genetic Mapping

**DOI:** 10.1534/g3.112.002428

**Published:** 2012-05-01

**Authors:** Y. Ronin, D. Mester, D. Minkov, R. Belotserkovski, B. N. Jackson, P. S. Schnable, S. Aluru, A. Korol

**Affiliations:** * Institute of Evolution, University of Haifa, Mount Carmel, Haifa 31905, Israel; † Department of Electrical and Computer Engineering, Iowa State University, Iowa 50014; ‡ Center for Plant Genomics, Iowa State University, Iowa 50011

**Keywords:** consensus genetic mapping, two-phase approach, synchronized marker ordering analysis, guided evolution strategy optimization

## Abstract

Numerous mapping projects conducted on different species have generated an abundance of mapping data. Consequently, many multilocus maps have been constructed using diverse mapping populations and marker sets for the same organism. The quality of maps varies broadly among populations, marker sets, and software used, necessitating efforts to integrate the mapping information and generate consensus maps. The problem of consensus genetic mapping (MCGM) is by far more challenging compared with genetic mapping based on a single dataset, which by itself is also cumbersome. The additional complications introduced by consensus analysis include inter-population differences in recombination rate and exchange distribution along chromosomes; variations in dominance of the employed markers; and use of different subsets of markers in different labs. Hence, it is necessary to handle arbitrary patterns of shared sets of markers and different level of mapping data quality. In this article, we introduce a two-phase approach for solving MCGM. In phase 1, for each dataset, multilocus ordering is performed combined with iterative jackknife resampling to evaluate the stability of marker orders. In this phase, the ordering problem is reduced to the well-known traveling salesperson problem (TSP). Namely, for each dataset, we look for order that gives minimum sum of recombination distances between adjacent markers. In phase 2, the optimal consensus order of shared markers is selected from the set of allowed orders and gives the minimal sum of total lengths of nonconflicting maps of the chromosome. This criterion may be used in different modifications to take into account the variation in quality of the original data (population size, marker quality, *etc*.). In the foregoing formulation, consensus mapping is considered as a specific version of TSP that can be referred to as “synchronized TSP.” The conflicts detected after phase 1 are resolved using either a heuristic algorithm over the entire chromosome or an exact/heuristic algorithm applied subsequently to the revealed small non-overlapping regions with conflicts separated by non-conflicting regions. The proposed approach was tested on a wide range of simulated data and real datasets from maize.

Numerous mapping projects have generated an abundance of mapping data. Consequently, many multilocus maps have been constructed using diverse mapping populations and marker sets for the same species. The quality of maps varies broadly between populations, marker sets, and applied software. As one would expect, there might be some inconsistencies among different versions of maps for the same organism. This calls for new efforts to integrate the mapping information and generate consensus genetic maps (MCGM) and integrate genetic and physical maps (Stam 1993; Klein *et al.* 2000; Kwitek *et al.* 2001; Williams *et al.* 2001; Wu *et al.* 2002; Menotti-Raymond *et al.* 2003; Yap *et al.* 2003; De Givry *et al.* 2005; Mester *et al.* 2006, 2010; Korol *et al.* 2009; De Keyser *et al.* 2010; and others). MCGM problem is even more challenging compared with mapping based on one dataset due to additional complications, namely, genetic and ecological differences in recombination rates and exchange distribution along chromosomes (Korol 2001), variations in dominance of the employed markers (Mester *et al.* 2003b), and different subsets of markers used by different labs. The maps created by different labs for an organism could contain different numbers of markers, and not all markers will be presented in each map. Markers presented in two or more maps can be referred to as *shared*, in contrast to *unique* markers presented in one map only. Four major approaches for consensus mapping can be considered:

(i) Reducing the consensus-mapping problem to single dataset ordering via constructing the synthetic distance matrix from all datasets (Wu *et al.* 2002). This approach does not use the notion of conflicting markers; the approximate EM algorithm is applied for optimization.
(ii) Graph-theoretic approach by looking for non-conflicting subset of markers via joint analysis of the previously constructed individual maps. This analysis is based on searching shared orders among the already constructed genetic maps from different mapping projects. For a conflicting pair of markers, the solution is obtained using heuristics that are based on some “voting” criterion (Yap *et al.* 2003; Jackson *et al.* 2008; Wu *et al.* 2008, 2011). For example, Jackson *et al.* consider a genetic map as a partial order, and the problem is solved by combining the graphs corresponding to individual partial orders and removing cyclic conflicts through the identification of minimum-feedback arc set. A combination of a heuristic algorithm and an exact solver are employed in the approach. This approach does not use the whole matrix of marker distances.
(iii) Using maximum-likelihood approach in two versions (De Givry *et al.* 2005): (a) if one assumes that the data from different sets represent a single map, *i.e.* same order and distances (this situation is referred to as *genetic merging*); or (b) if the same order is assumed, but recombination rates differ between the mapping populations (this situation is referred to as *order merging*).
(iv) Reducing consensus mapping to a specific (constrained) version of the traveling salesperson problem (TSP) that can be referred to as “synchronized TSP.” To solve the problem, we search for the best multilocus order corresponding to minimum of weighted sum of map lengths among non-conflicting orders, testing only those orders that fit the condition “shared markers must be in shared order” for any subgroup of mapping populations (Mester *et al.* 2003b, 2006, 2010; Korol *et al.* 2009). This approach uses the whole matrix of marker distances for each mapping population included in the analysis. A combination of heuristic and exact algorithms is used in the approach.

These approaches to solve MCGM were implemented as software packages: (i) JoinMap (http://www.kyazma.nl/index.php/mc.JoinMap/); (ii) MergeMap (http://138.23.178.42/mgmap); (iii) CarthaGene (http://www.inra.fr/internet/Departements/MIA/T//CarthaGene/); and (iv) MultiPoint (http://www.multiqtl.com/). Here, we introduce new algorithms for the forth approach, which are more effective than our previously published variant (Mester *et al.* 2006). The algorithms were implemented in MultiPoint-consensus software (http://evolution.haifa.ac.il/images/stories/korol_lab/Programs/MultiPointConsensus_DemoG.zip), validated on simulated datasets and illustrated on real data.

The major points of our approach concern the chosen optimization criteria for searching for the best multilocus order and representation of the integral map resulting from consensus analysis of multiple datasets. For building consensus (non-conflicting) maps, we employ methods of conditional minimization (the condition is “shared order for shared markers”) of the weighted sum of individual chromosome lengths. In using this approach, we take advantage of high efficiency of our hybrid heuristics for discrete optimization. In building the integral map, we take into account the possibility of local map uncertainty (branching) caused by the presence of unique (set-specific) markers. This uncertainty, inescapable in many cases, is ignored by some mapping packages in which the integral map is based on averaged recombination distances. An interesting perspective to address this problem is to take advantage of the comparative genomic approach (Faraut *et al.* 2007) based on the evolutionary conservation of chromosome segments between related species.

## Models, Methods, and Algorithms

### Models and methods

In our approach, the optimal consensus order of shared markers is defined by searching the minimal total length of non-conflicting maps of the chromosome. The employed criterion is based on the sum of recombination rates across adjacent segments and is widely used in map construction for single-population data (see Molinari *et al.* 2009 for review). Unlike the analysis of a single dataset, in consensus mapping the optimization is conducted simultaneously on all sets or some subset of all mapping populations. The additional request is non-conflicting order of shared markers. Let *W_i_* be the set of all markers of the analyzed chromosome of the *i*^th^ population (dataset). Then, _$W=\cup W_{i}$_ is the set of all markers of the consensus mapping problem. Consider markers that appear in at least one pair of mapping populations. The set *R* of such markers is referred to as *shared markers*. Correspondingly, *V = W-R* is the set of markers that appear in only one of the mapping populations and can be called unique markers.

Denote ***G*** as the set of all possible partial orders *g* of shared markers and by *U*(*i*, *g*(*i*)) the set of all permutations of unique markers of the *i*^th^ dataset relative to order *g*(*i*)⊂*g* of shared markers of this set. An optimal partial order g* of shared markers can be defined as one from the set ***G*** minimizing the sum

$$S\left(g\right)=\sum_{i}w_{i}\min_{u\varepsilon U\left(i,g\left(i\right)\right)}S_{i}\left(g\left(i\right),u\right)=\sum_{i}w_{i}S_{i}\left(g\left(i\right),u_{i}\left(g\left(i\right)\right)\right).$$

Here *S_i_* is the sum of all recombination rates along the map for the *i*^th^ set (population) of the analyzed chromosome; order *u_i_*(*g*(*i*)) from set *U*(*i*, *g*(*i*)) is the one that provides the minimum of *S_i_*. Coefficient *w_i_* is the weight of *i*^th^ set in the optimization criterion that can characterize the quality or reliability of the data. This criterion can be modified to take into account the variation in the quality of original datasets (*e.g.* population size and/or marker quality).

Generally, our approach is based on joint analysis of raw mapping data using a two-phase analytical scheme. In phase 1, multilocus ordering combined with iterative resampling (to evaluate the stability of marker orders) is performed separately for each dataset (Mester *et al.* 2003a). In phase 2 we employ two methods, *global* and *local*, for solving the problem. In the global method, consensus mapping is conducted by reducing the problem to synchronized TSP (Mester *et al.* 2006). In the local method, the regions of conflicting order are defined and for each such region the analysis is conducted using criterion (1). If a conflicting region is small, an effective scheme of full enumeration is proposed; otherwise, we employ the heuristics of synchronized TSP. The results of local analysis can also be used as starting point for the global analysis. The main reason for using local analysis is the complexity of optimization for TSP with constrains. Even a single constrain may request more sophisticated algorithms and a considerable increase in computation time (sometimes by orders of magnitude; see TSP-like benchmarks and results in http://www.sintef.no/Projectweb/TOP/Problems/VRPTW/Homberger-benchmark/).

In case of MCGM, ordering of shared markers across all analyzed datasets tremendously increases the computational complexity compared with the standard single-set TSP-like formulation, which itself is computationally challenging (Weeks and Lange 1987; Falk 1992; Mott *et al.* 1993; Schiex and Gaspin 1997; Hall *et al.* 2001). As mentioned earlier, we employ in our approach the criterion of total sum length of the chromosome maps or the criterion of the total length of each region of conflict. These criteria can be modified to take into account variation in the quality of original datasets (*e.g.* population size and/or marker quality). Moreover, various other criteria employed for single-map construction can be adapted to the proposed here synchronous ordering approach.

### Algorithms for consensus mapping

For consensus mapping (phase 2), we developed algorithms for the aforementioned local and global analysis. The local method includes: (a) defining the regions of local conflicts based on pairwise conflicts and (b) the algorithm of full enumeration of all non-conflicting orders of shared and unique markers in the defined conflict region (see below) to find the solution (local order with the best value of the criterion) in reasonable time. The algorithm for the global method uses special heuristics for global discrete optimization of synchronized TSP for all markers (unique, shared conflicting, and shared non-conflicting) (Korol *et al.* 2009; Mester *et al.* 2010). Here the process of optimization is conducted along the entire chromosome using the solution obtained with the local method as an initial point. It is noteworthy that the global algorithm may be very useful in the local method when the size of the conflicting region is too large to employ the optimization based on full enumeration.

**Figure 1:**
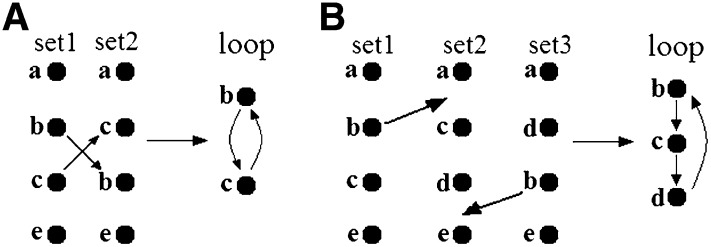
Examples of local conflicts: (A) pairwise conflict and (B) higher-order conflict.

**Figure 2:**
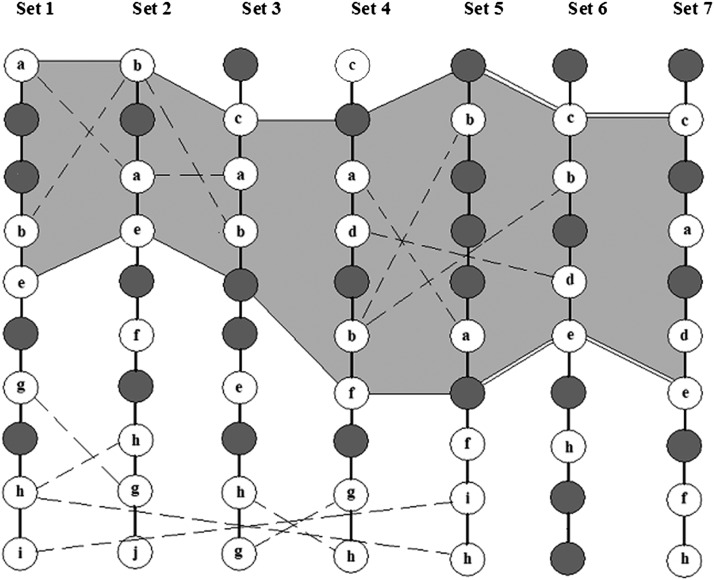
Stepwise determination of conflict regions. White circles denote shared markers, and gray circles denote unique markers. The figure illustrates the process of delineating conflict regions based on overlapping regions of pairwise conflicts. Conflict between markers a and b includes the first five sets; the conflict region is bordered by a single line. Conflict between b and d starts in the first region (at set 4) and extends until the end (set 7) (its part outside the first region is marked by double lines). The whole conflict region is highlighted in gray.

**Figure 3:**
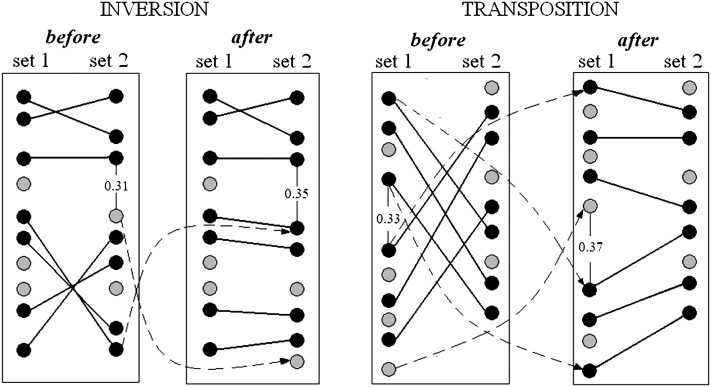
Inversion and transposition transformations in consensus analysis. Black and gray circles represent shared and unique markers, respectively. Shared markers with the same name are connected with solid lines. The left part of the figure illustrates the operation “inversion”: due to a large interval in set 2 (recombination rate ∼0.31), the distal part of the map in set 2 is in conflict with the one in set 1. Operation “inversion” returns a nonconflicting order. Similarly, in the right part of the figure, a source of the conflict is the interval with loose linkage in set 1. Operation “transposition” returns a nonconflicting order.

Let us consider in more detail the developed algorithms. After getting the non-synchronized solutions based on independent mapping for each dataset (single-population mapping), conflict regions are formed by the analysis of all pairs of the resulting individual maps. Each conflict region contains shared conflicting and non-conflicting markers, and some unique markers. The remaining non-conflicting shared markers *between* the conflict regions are considered as “frozen” anchors during the solution process for each conflict region (hence, only conflict region markers participate in the optimization process). This version of the algorithm significantly reduces the computation time. Moreover, for certain sizes of conflict region, an exact solution can be obtained. The sequential steps of the analysis include:

1. Building skeleton maps for each of the initial datasets using the methodology described in Mester *et al.* (2003a), Korol *et al.* (2009), and Ronin *et al.* (2010).
2. Revealing conflicting shared markers for each pair of chromosomes (Figure 1A).
3. Integration of the results on detected pairwise conflicts (across all involved datasets) allows defining the conflicting regions separated by non-conflicting regions (Figure 2).
4. In case of a large interval between neighboring markers, the corresponding map may display an “inversion” or “transposition” of a large segment resulting in multiple conflicts with other maps. In such a case, before starting the main consensus procedure, we employ heuristic rules of transposition and inversion to get a better initial point for consensus ordering (Figure 3).
5. Evaluating the computation complexity of the conflict regions. On this stage, a crude estimate of the computation time needed to resolve the conflict by testing all possible permutations is provided. If the needed time is not affordable, the alternative is to use heuristic optimization.
6. Resolving the conflicts in each conflict region by synchronous optimization. This means that the allowed trial solutions must fit the following condition: for the considered chromosome, the map order in none of the mapping populations is in conflict with the order in any other mapping population. Obviously, for such a formulation, one may take advantage of parallel computations.
7. Detecting and resolving higher-order conflicts and loops, which are much less frequent than pair conflicts (Figure 1B).

As a conflict region, we imply a set of segments of the maps for the mapping populations that include shared and unique markers and fit the conditions: (a) unique markers are flanked by or are adjacent to the shared markers and (b) at least for one pair of populations, two or more shared markers from the region appear in conflict order. The procedure of defining conflict region is illustrated in Figure 2. The analysis of several datasets may reveal a few conflict regions separated by regions of no conflict. In Figure 2, the process of delineating a conflict region is based on overlaps of pairwise conflicts. The conflict between markers *a* and *b* includes the first five sets; the conflict region is bordered by a single line. A second conflict, between *b* and *d* markers, starts at set 4 and extends until set 7. In defining the boundaries of the conflict region, we employ the following rules: (i) the size of the region depends on the conflict markers, where a conflict marker can coincide with the boundary only if it is simultaneously the end marker of the linkage group (in the example in Figure 2, conflict markers *а* and *b* coincide with the end in sets 1 and 2, respectively); (ii) in all other cases, boundary marker for the conflict region may be either a non-conflict shared marker or a unique marker; and (iii) depending on the width of the conflict region, we employ the exact or heuristic optimization algorithm.

Local algorithm of consensus analysis, in contrast to global analysis, applies only to the conflict region. With several regions, the conflicts are resolved successively in each such frame. A conflict region should include at least a pair of shared markers, the order of which in at least one set is opposite to the order in other sets. The conflict region of the entire set of mapping populations should include the considered pair of conflicting markers as well as all markers in between this pair, either shared or unique. In turn, the shared markers in the conflict region can also be in conflict with other markers. This conglomerate of pairwise conflicts makes up a conflict region that can also be extended by including neighbor markers from both sides that do not create any conflict (see Figure 2). This extension is needed because during the process of resolving the conflict(s) within the conflict region, the flanking markers are frozen. Note that if one of the ends of the conflict region coincides with the end of the linkage group, then only one of the ends (the internal one) remains frozen during the optimization.

The computation complexity and the efficiency of the proposed scheme of exact solution can be illustrated by the following calculations. Consider a conflicted interval of *k* shared conflicted markers bounded by a pair of non-conflicted markers. Let *n* be the number of unique markers in one dataset. In this situation, the total number of different orders is (*k*+*n*)!. For each order, a sum of *k*+*n*+1 elements should be calculated. According to the proposed scheme, in the first stage we prepare a table of 2*^n^*((*k*+2)(*k*+1)/2−1) optimal orders for each of the possible subsets of *n* unique markers in the intervals bounded by the pair of shared markers (for each possible pairs of *k*+2 shared markers, excluding the pair of bounded markers). The worst case corresponds to situations where all of *n* unique markers are situated in one interval of shared markers (the number of possible orders in this situation is *n*!). In the second stage, we test all *k*!(*k*+1)*^n^* combinations of the orders of *k* shared markers and arrangements of *n* unique markers among *k*+1 resulting intervals of *k*+2 shared markers. For each such combination, we calculate the criterion value as a sum of *k*+1 elements of the table prepared in the first stage. This tabulation-based method significantly reduces the computation time for finding the exact solutions. For example, in a situation with *k* = 4 and *n* = 10, using this scheme reduced the calculation time about 325-fold compared with the time one would need for consequent testing of all possible permutations. In fact, we employ a scheme of exhaustive permutations, where with increasing number of markers, the majority of marker orders can be skipped due to the preliminary computations saved in the memory. With *k*+*n* < 15 (of which the number of either shared *k* or unique *n* is <11), the exact solution can be obtained in less than an hour.

The main idea of solving MCGM using the global method is to generate possible partial orders *g* of shared markers ***R*** and evaluate them by means of criterion of minimal total length *S*(*g*) (equation 1) taken across all datasets of the problem. The optimal consensus order of shared markers is defined by searching minimal total length of non-conflicting maps of the chromosome. Unlike the analysis of a single dataset, where the order is optimized based on the sum of recombination distances for one map only, here the optimization is conducted simultaneously on all sets or on some subset of all sets. Each dataset *i* contains some shared markers ***R***(*i*)ε***R*** named as *anchors*, and some unique (set-specific) markers *V*(*i*). Here computing the criterion (1) includes determination for each dataset *i* the optimal order of unique markers *V*(*i*) using the order of shared markers as anchors. The mapping problem is reduced to a TSP with special restriction on order of anchor markers. We do not know about any existing specific method for the TSP that can be directly applied to this specific sophisticated situation. Therefore, for searching the solution to the problem, we decided to use a meta-heuristic, guided evolution strategy (GES). It is a hybrid heuristic algorithm that combines the power of guided local search (Voudouris 1997) and evolution strategy (ES) algorithms (Mester *et al.* 2003a, 2007, 2010). Our GES algorithm uses the basic ideas of ES (Rechenberg 1973) and (*μ*, *λ*)-strategy (Schwefel 1977), which were supplemented with new elements. Namely, we extended the ES algorithm by including variable size mutations, random mutation schemes, and a heuristic algorithm that defines the “reasonable” places of mutation (for large-scale problems) (Mester *et al.* 2010). GES successfully solves some constrained discrete optimization TSP-like problems (Mester and Braysy 2005, 2006; http://www.sintef.no/Projectweb/TOP/Problems/VRPTW/Homberger-benchmark/), classic TSP (Mester *et al.* 2010), and genetic mapping problems formulated in TSP-like terms with the anchor constraints (Mester *et al.* 2004, 2006, 2010; Korol *et al.* 2009). The GES algorithm was adapted to solve synchronized optimization problems when the exact algorithm of the local method is not effective due to the large size of the conflict region. Namely, GES was strengthened by three additional local search procedures for working with anchor-marker constrains: “Reinsert,” “Reverse-Reinsert,” and “Exchange 1*1” (Braysy and Gendreau 2001). A new *multi-parametric generator* was developed to create random orders *g* of shared markers. In our scheme, six parameters define mutation of current best solution *g*^best^ (Mester *et al.* 2010).

For getting the exact solution of MCGM via the global method we must generate all (*n*!/2) possible orders of shared markers in *g*. Clearly, it does not make sense trying all generated orders *g* of shared markers with respect to criterion (1), which includes both shared and unique markers, because the majority of orders will be very far from the optimal order. For estimating the quality of generated order *g^t^* at step *t*, the second (*skeleton*) criterion, is used. It is computed as total map length *S*^(^*^t^*^)^_shared_ = *∑_i_ w_i_ S_i_*(*g^t^*), taking much less time. Note that the total map length *S*^(^*^t^*^)^, referred to as *main criterion*, is calculated according to eq.1, *i.e.* includes both the shared and unique markers. During the optimization process, all current *S*^(^*^t^*^)^_shared_ and corresponding best *S*^(^*^t^*^)^ values are stored in a special list. The values of *S*^(^*^t^*^)^_shared_ and *S*^(^*^t^*^)^ are highly correlated, although the extremes do not necessarily coincide (*i.e.* minimum of *S* may not correspond to the minimum of *S*_shared_). Therefore, checking the main criterion for each generated order *g* of the shared markers does not seem to be a good idea. We compute the criterion *S*^(^*^t^*^)^ only for those *g^t^* that satisfy the condition *S*_shared_(*g^t^*) ≤ *q*, where parameter *q* is the threshold value of *S*_shared_ (Mester *et al.* 2010). After a new successful step of optimization (that provides an improvement of *S*^(^*^t^*^)^), the new (*S*^(^*^t^*^)^_shared_, *S*^(^*^t^*^)^) pair is included to the list and *q* is recalculated. Changing *q* along (and dependent on) the optimization trajectory can be considered as a learning process that allowed reducing 20- to 100-fold the computation time.

Two starting points are used in the global method: g* (optimal solution obtained by the local method) and quick skeleton solution g** (minimum of *S*_shared_) defined by the GES algorithm (Mester *et al.* 2006). Main steps of the global method can be presented as:

1. Define initial solution
  $\begin{array}{l} g^{0}=\text{best}\;\text{of}\;\left(g*,g**\right)\\ g^{\text{best}}=g^{0};t=0\\ S\left(g^{\text{best}}\right)=\sum_{i}w_{i}\min_{u\varepsilon U\left(i,g\right)}S_{i}\left(g^{\text{best}},u\right)=\sum_{i}w_{i}S_{i}\left(g^{t},u_{i}\left(g^{\text{best}}\right)\right) \end{array}$
2. *t* = *t*+1
3. Generate new *g^t^* on *g*^best^ via muti-parametric generator
4. If *S*_shared_(*g^t^*) ≤ *q* then
  {
5. Define *S*(*g*^*t*^) via GES algorithm
6. If *S*(*g^t^*) < *S*(*g*^best^) then *g*^best^ = *g^t^*
  }
7. If not finished go to step 2

To test the efficiency of GES, we simulated single mapping populations with up to 1000 markers per chromosome, whereas for testing constrained optimization problems in consensus mapping, our simulations included multiple populations (up to 16) with up to 100 markers per population with different proportions of shared markers.

**Figure 4:**
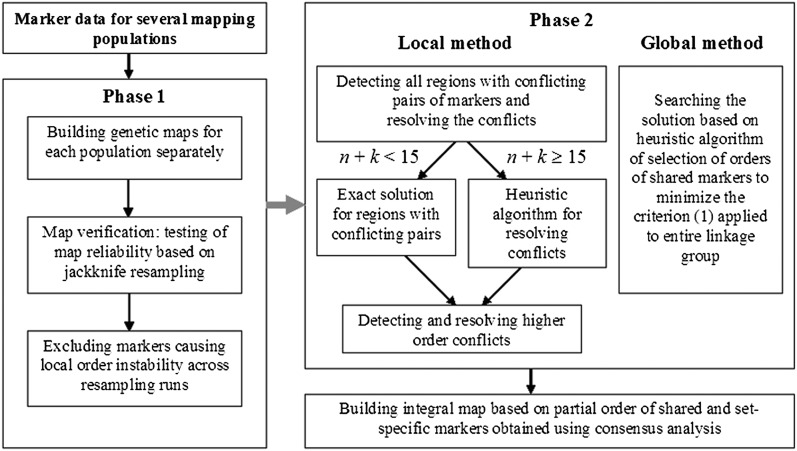
The general scheme of the proposed two-phase approach of consensus mapping.

The general scheme of the proposed two-phase approach of consensus mapping analysis is presented in Figure 4.

## Results

### Analysis of simulated data

Simulated data are the main instrument allowing the objective comparison of different analytical schemes of consensus analysis. It is highly desirable to imitate as many as possible features of real data that may be important for the solution process and affect the solution quality. Many of these features are the same as in multilocus mapping for single-population situations (*e.g.* Mester *et al.* 2003a, b), but there are also some differences:

- Differences between the populations in the rate and chromosomal distribution of recombination events
- Variation in interference (positive and negative)
- Various levels of missing data
- Scoring errors varying among the loci and populations
- Variation in the proportions of shared markers

The main quality characteristic in our previous single-population analysis was the recovery coefficient (Mester *et al.* 2003a). This characteristic can also be applied in simulation analysis of consensus mapping analysis, with a small modification: the recovery coefficient should be considered for shared markers.

Before moving to the results obtained for simulated data, a short explanation of employed marker names should be provided. In each set, markers located sequentially along the chromosome were enumerated sequentially. The names of shared markers look like *mar*1, *mar*2, *mar*3, etc., and the unique marker names also include the set number. Thus, markers located consequentially along chromosome for *i*^th^ set can be represented as *mar*1, *mar*2*_i*, *mar*3*_i*, *mar*4, *mar*5, *mar*6*_i,…*, where the 2^nd^, 3^rd^, and 6^th^ markers are unique to set *i*.

In the first two examples, we compare the effects of missing data and small sample size with the number of conflicts and quality of consensus mapping. Namely, the population size in the first example was 150, but due to missing data in the first eight sets, the average size per marker pair was 56; in the second example, the sample size for the first eight sets was 50 and no missing data were simulated. The simulated datasets used in this article can be downloaded (http://evolution.haifa.ac.il/images/stories/korol_lab/Programs/SE.rar).

#### Example 1.1:

Example 1.1 includes single-chromosome data on 16 F_2_ populations each with sample size 150 genotypes scored for 50 codominant markers (supporting information, File S1, example 1.1). In 75% of the cases, the interval length was generated as an evenly distributed variable in range [1, 4] cM, and in 25%, in range [4, 20] cM. For the first eight variants, 40% of scores were simulated as missing data, whereas in the remainder 8 variants the missing scores comprised 20%. In addition, in 10% of individuals, 20% of loci of the first group and 80% of the second group were simulated as misclassified. Namely, for loci with misclassification, 5% of heterozygous genotypes H = AB are classified as AA and 5% as BB; and 10% of homozygotes AA and 10% of BB are classified as AB. The results of consensus analysis of the data are shown in Table 1. Here, as well as in Table 2, Table S1, and Table S2, we show the map length for each population obtained based on individual analysis of the population data (Ind) and based on joint (consensus) analysis (Cons); the order of shared markers is also shown. Marker names are represented by consecutive numbers in the chromosomes, so that the true order corresponds to monotonically increasing numbers. Places with wrong order are highlighted in gray; for such places, left parts of the columns correspond to individual ordering, whereas right parts correspond to consensus mapping. We can see that in the vast majority of cases consensus analysis corrects the local deviations from the true order.

**Table 1:** Effect of high missing data on map quality in individual and consensus mapping (simulated data of example 1.1)

Set	1		2		3		4		5		6		7	8		9		10		11	12		13	14		15		16	
Missing %	40															20													
Ind (cM)	256		302		290		227		348		288		294	259		616		589		595	556		553	613		592		484	
Cons (cM)	261		324		295		240		351		299		294	269		621		593		595	558		553	618		596		491	
	**3**	1	**3**	1	2		3		1		2		1	**3**	2	5		3		2	1		6	2		**5**	2	3	
	**1**	3	**1**	3	3		10		2		6	5	2	**2**	3	7		4		3	5		8	4		**2**	5	4	
	5		8		5		12		5		5	6	3	6		10		5		4	6		10	11		6		5	
	9		9		8		**15**	14	6		7		4	8		13		8		6	**8**	7	12	14		10		6	
	10		**12**	10	10		**14**	15	8		8		5	11		17		11		7	**7**	8	13	**17**	16	12		7	
	12		**11**	11	12		17		9		9		6	12		19		**15**	14	10	12		16	**16**	17	15		12	
	13		**10**	12	14		21		15		**12**	10	7	14		**24**	21	**14**	15	11	14		17	19		18		13	
	14		13		**17**	16	22		16		**10**	12	10	18		**21**	24	17		13	15		18	20		19		14	
	15		**15**	14	**16**	17	23		**18**	17	14		11	19		30		18		16	17		19	22		20		16	
	16		**14**	15	18		**25**	24	**17**	18	16		12	22		31		21		18	**21**	18	21	25		**22**	21	17	
	19		17		21		**24**	**26**	19		17		16	**24**	23	33		23		20	**18**	20	23	29		**21**	22	19	
	21		19		24		26	**25**	23		18		17	**23**	24	35		24		21	**20**	21	25	31		27		20	
	22		22		26		27		25		19		19	28		42		27		22	24		28	36		28		**26**	26
	23		23		28		30		31		22		20	29		43		28		26	27		33	37		32		**25**	25
	29		**25**	24	29		32		33		23		21	30		46		30		28	30		34	39		34		28	
	30		**24**	25	30		34		36		24		22	31		47		39		30	36		35	41		35		29	
	**32**	31	28		31		37		38		**26**	**26**	24	33		50		42		31	37		36	40		38		30	
	**31**	32	29		**34**	33	**46**	44	40		**27**	**25**	27	34				46		32	40		37	42		39		31	
	34		31		**33**	34	**45**	45	**47**	41	**25**	27	28	38				**48**	47	35	41		38	43		**42**	40	33	
	35		33		38		**44**	46	**45**	43	28		32	**41**	40			**47**	48	38	43		41	47		**40**	42	**35**	34
	37		34		40		47		**46**	45	29		33	**42**	41			50		42	44		45	49		43		**34**	35
	39		36		**49**	45	48		**43**	46	30		35	**40**	42					45	45		46	50		**50**	48	38	
	41		**41**	39	**50**	49			**41**	47	31		37	45						46	46					**48**	50	**40**	39
	44		**39**	41	**45**	50					34		38	46						47	47							**39**	40
	50		47								36		41	47						49	49							43	
											37		43	48							50							44	
											38		44	49														45	
											39		48															46	
											**41**	40	50																
											**40**	41																	
											42																		
											43																		
											44																		
											45																		

**Table 2:** Effect of reduced sample size (comparable with missing data in example 1.1) on map quality in individual and consensus mapping (simulated data of example 1.2)

Set	1		2		3	4		5		6	7		8		9		10		11		12	13		14		15		16	
Sample size	50														150														
Ind (cM)	304		238		231	466		233		288	310		348		652		613		552		551	475		597		528		579	
Cons (cM)	310		238		231	469		233		288	310		347		659		619		554		551	482		603		530		579	
	1		1		1	2		4		1	1		1		1		1		**2**	1	3	6		**5**	1	1		2	
	3		4		5	3		5		2	3		3		2		3		**1**	2	5	7		**1**	3	**3**	2	3	
	7		**7**	6	10	6		6		3	4		8		**5**	4	4		3		6	8		**4**	4	**2**	3	7	
	8		**6**	7	11	**13**	12	7		4	13		9		**4**	5	10		5		8	9		**3**	5	5		8	
	9		8		13	**12**	13	9		7	14		10		7		**15**	14	6		9	11		7		6		**12**	10
	10		9		14	14		10		11	15		11		8		**14**	15	8		11	**14**	13	8		7		**10**	12
	11		**13**	11	15	15		13		18	16		13		9		19		13		12	**13**	14	11		9		13	
	14		**11**	13	16	16		16		20	17		14		10		22		16		13	19		**14**	13	12		14	
	18		16		17	17		17		21	21		17		12		24		17		14	20		**13**	14	13		16	
	**21**	20	23		18	18		19		22	23		18		13		25		26		16	23		15		14		17	
	**20**	21	24		20	**22**	20	20		23	23		19		18		28		34		17	**25**	24	16		15		19	
	23		26		22	**21**	21	22		25	25		21		21		24		36		23	**26**	25	17		20		20	
	24		29		23	**20**	22	24		26	27		22		22		**32**	30	39		26	**24**	26	18		22		21	
	25		30		24	23		25		27	29		23		23		**30**	23	40		29	28		19		24		24	
	27		33		25	24		27		29	35		24		**26**	24	33		**42**	41	30	31		20		28		27	
	30		35		28	25		29		32	36		25		**24**	26	36		**41**	42	33	33		**26**	25	30		29	
	31		37		30	26		30		33	**38**	37	26		29		39		44		34	34		**25**	26	32		31	
	32		39		32	30		34		35	**37**	38	27		38		40				36	35		30		33		32	
	35		41		34	**33**	32	33		38	39		**30**	28	39		41				37	39		31		36		33	
	37		43		35	**32**	33	36		40	**43**	41	**28**	30	40		43				38	41		33		37		34	
	38		47		36	34		37		41	**41**	43	31		41		44				40	42		34		39		35	
	43		50		37	35		**39**	38	42	47		34		44		46				41	43		36		40		36	
	45				41	43		**38**	39	44	48		35		46		48				42	44		**42**	39	41		38	
	48				44	46		40		45	49		36		49		50				47	45		**39**	42	42		41	
	49				45	48		41		47			43		50						50	48		43		**45**	44	44	
	50				46	49		42		48			46									49		46		**44**	45	45	
					48	50		44		50			47									50		47		47		46	
					49			46					48											50		49		48	
					50			48					50															49	
								49																					
								50																					

Ind (cM) is chromosome length obtained under individual analysis of each datasets (in phase 1). Cons (cM) is chromosome length obtained under consensus analysis (in phase 2). Each column represents shared markers of a dataset (mapping population). The numbers of shared markers correspond to their order in the simulated maps (including the sets-specific, or unique, markers, although the latter are not shown in the table). In some cases, a column of a set includes two rather than one marker number. The left number is always highlighted by a shaded background to note that individual ordering (phase 1) resulted in wrong positioning of this marker; the right number displays the situation after consensus ordering: shaded background notes that consensus ordering did not succeed whereas unshaded background means that consensus analysis returned the correct order.

#### Example 1.2:

In contrast to the previous example, no missing data were in the first eight populations, but their sample size was only 50 per population, *i.e.* 1/3 of the sample size of the second group of eight populations (File S2, example 1.2). All other parameters were as in example 1.1. The results of consensus analysis are shown in Table 2.

In both examples, the increased level of scoring errors resulted in inflation of map lengths in the second group of populations. The differences between the results obtained for examples 1.1 and 1.2 can easily be explained. In example 1.1, due to 40% of missing data, only 56 genotypes remain, on average, for estimation of pairwise distances between markers, *i.e.* nearly the same as in the first eight populations of example 1.2. In example 1.2, individual analysis of each dataset resulted in maps with 28 cases of deviation from the simulated order of shared markers (in total) across the first eight datasets; in example 1.1 the number of such cases was nearly 2-fold (52). Thus, missing has a more influential effect on the quality of multilocus ordering than an equivalent decreasing of the sample size. Nevertheless, in both examples, consensus analysis managed to recover the true order, except for one case in example 1.1.

#### Example 2.1:

The sample size for each of the 16 populations was 100. The first 8 populations were simulated with no missing data, whereas 20% of missing data were simulated for the remaining 8 populations (File S3, example 2.1). Moreover, zero level of scoring errors was simulated in the first group and 10% of error alleles at 75% of loci were in the second group. The absence of missing scores and errors in the first group of populations resulted in exact individual ordering of each chromosome, unlike the second group with more than three wrong orders per set (Table S1). Two rounds of consensus analysis were conducted. In the first, equal weights were applied to all populations in the optimization criterion. In all sets, except one, erroneous local neighborhoods caused by missing data and marker scoring errors were corrected via consensus analysis. Only one segment from variant 13 remained unfixed and, during consensus analysis, caused an ordering error in set 1. Thus, only one out of 28 segments remained uncorrected. This exemplifies a situation in which consensus analysis results in an increased order quality compared with the wrong solutions obtained for individual sets due to the noise effects. It is noteworthy that this result was obtained without using an important resource: the information about the quality of scoring data.

Obviously, one would expect the results obtained with noisy data to be less accurate; hence, it makes sense to decrease the contribution of more noisy data to the optimization criterion compared with less noisy data. The simplest indicator of data quality may be just the map length *L_i_* for the corresponding *i*^th^ dataset analyzed individually (before consensus step). Indeed, with other things being equal, the map length will inflate monotonically with the level of scoring errors. This effect can be easily seen in Table 1, Table 2, Table S1, and Table S2, in which noisy variants have, on average, 2-fold longer maps than the “pure” variants. To reduce the impact of noisy variants to the consensus solution, one can conduct a second round of calculations using 1/*L_i_* as weights. In our example, when 1/*L_i_* weights were employed, the less noisy (more informative) dataset 1 overweighed the effect of the more noisy dataset 13, resulting in a correct order in the region of markers 18–19.

#### Example 2.2:

Example 2.2 is based on eight simulated F_2_ mapping populations each with *N* = 100 genotypes and *m* = 50 markers. In the first four sets, there were neither missing nor scoring errors, whereas in the remaining four sets 20% of missing scores across all marker loci and 10% erroneous scores across 75% of loci were simulated. In the example, after phase 1 analysis, we observed altogether 17 conflicts; all of these were resolved in phase 2. For illustration, let us consider one of these conflicts in more detail, for *m25* and *m26* loci, involving four out of the eight sets (Table S2). The conflict is caused by inconsistency of the optimal orders of these markers in sets 5–7 with the optimal order (*i.e.* the correct one, coinciding with the simulated order) of these markers in set 1. For these four sets, *S_i_* values at the solution orders resulted from separate analysis of individual sets were 244, 552, 578, and 542 cM, respectively. The two conflicting orders are shown in Figure S1. With the erroneous solution, the contributions of the defined conflict region to *S_i_* are equal to 0.25, 0.32, 0.81, and 0.47, respectively (the non-weighted sum = 1.84, see Figure S1A). With the correct solution, the contributions were 0.18, 0.32, 0.81, and 0.51, respectively (sum = 1.82, see Figure S1B). Clearly, the second solution is preferable: its criterion value is smaller by 0.02. Using non-equal weights *w_i_* decreases the contribution of the erroneous solution to optimization criterion *S* because missing data and scoring errors resulted in an elongation of maps in datasets from the second group. This increases the difference between the correct and erroneous solutions about 4-fold. Clearly, the simple “voting” approach would not work here because only one out of the four sets displayed the correct order between *m25* and *m26*.

### Analysis of real data

We illustrate here the developed methodology and algorithms in consensus mapping example on maize. Two groups of data were analyzed, one with six BC populations and the other with 24 RIL populations (http://www.panzea.org/db/gateway?file_id=NAM_map_and_genos). The results for both groups of data include correction of the individual maps based on consensus analysis and building of integral maps.

#### Maize six BC populations:

The major step in our multilocus mapping is building a framework (skeleton) map. With this approach, the skeleton map is cleaned from markers that fit one of these conditions: (i) are absolutely linked; (ii) violate monotonic growth of recombination with its subsequent neighbors; (iii) have unstable location relative to other markers (as detected by jackknife resampling). During consensus mapping, the first step is also building skeleton maps for each dataset, but now we should retain as many shared markers as possible. Thus, on the first step, we delete only unique markers that fit one of the three conditions and each of the shared markers that is absolutely linked to the same shared marker in other sets. This allows obtaining a richer integral map at the end of the analysis. Below we present the results on consensus analysis of six BC populations (Set1–Set6). Comparing the lengths of maps constructed based on individual and consensus analysis allows judging the quality of the data.

**Figure 5:**
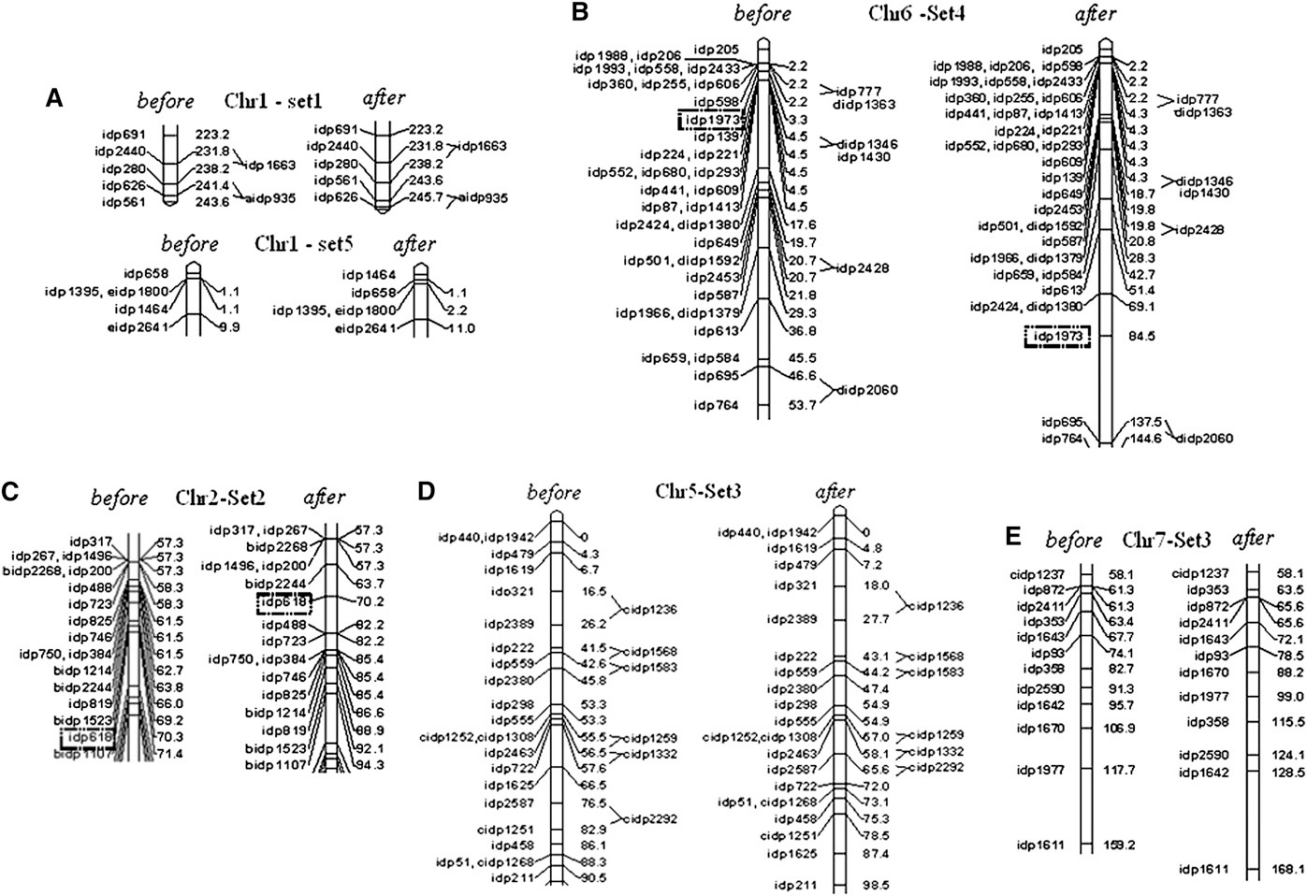
Examples illustrating the “cost of consensus” (based on joint analysis of six BC maize populations): (A) low cost when there is no need in removing markers to reduce the cost; (B, C) high cost justifying removal of one marker in each case; and (D, E) moderate cost caused by marker pairs extending the map (deleting these markers reduced the segment length 2-fold).

**Figure 6:**
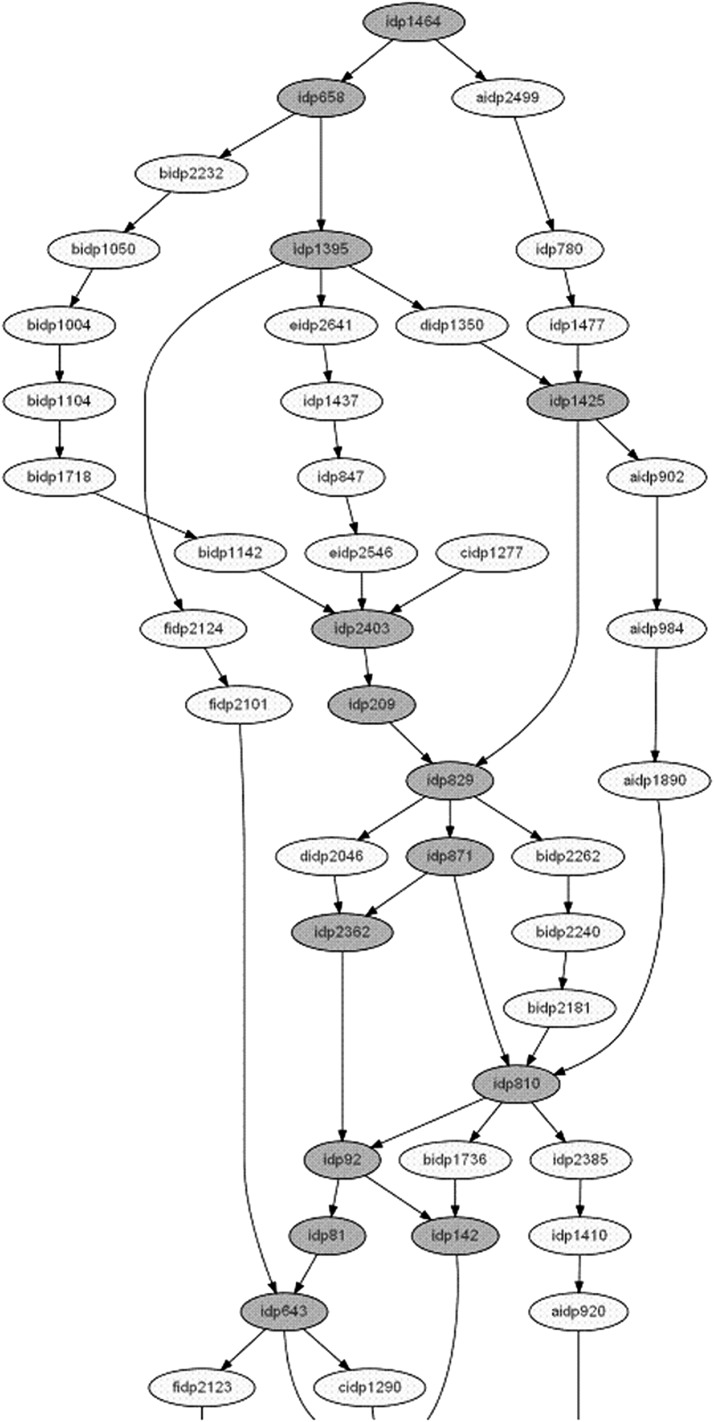
A fragment of the integral map of chromosome 1 (based on data of six BC mapping populations of maize). Dark nodes denote shared markers, and light nodes denote unique markers.

Three situations can be considered: (a) no difference between the lengths, implying no conflicts between the individual maps (like in chromosome 9, Table S3); (b) small differences, which point to local conflicts resolved by consensus analysis with a minor elongation of the map (this situation was found in most chromosomes for most of the sets; see, for example, chromosome 1 in sets 1 and 5, Table S3, and Figure 5A); and (c) larger difference, indicating a considerable conflict, as found for chromosome 6. The situation of chromosome 6 in set 4 before and after consensus analysis is presented in Figure 5B. Considerable inflation of the map length after consensus analysis may reflect a low quality of some of the markers or be a result of a real biological reason (*e.g.* inversion or transposition). In any case, the *cost of consensus* in a situation like the one found in chromosome 6 (set 4) may be too high, calling for detection and removing of the “responsible” marker from the set in which its position in the initial map seriously differs from that in other datasets. In the considered example, the map expansion was caused by the presence of marker *idp*1973. Table S3 shows the results for map lengths for all 10 chromosomes, including the corrected situation for chromosome 6 after removing marker *idp*1973 from set 4. We can see a good correspondence between the results of individual and consensus mapping. In addition to the previously mentioned case of “high cost” of consensus, a few additional cases may deserve similar consideration: Chr2-Set2 (144.8–167.7); Chr5-Set3(158.3–166.4); and Chr7-Set3(191.7–200.6) (see Figure 6, C–E, and Table S3). For Chr2-Set2 (Figure 5C), removing marker *idp*618 solves the problem of the high cost of consensus caused, actually, by two markers, *idp*618 and *idp*488 involved in non-local conflict in sets 1 and 2, and separated by 21 cM in set 1 and 11.2 cM in set 2. For Chr5-Set3 (Figure 5D), markers *idp*1625 and *idp*2587, which caused an expansion of *idp*722-*сidp*1251 from 2.1 cM to 25.3 cM, were removed. Similarly, for Chr7-Set3 (Figure 5E), markers *idp*1670 and *idp*1977, which caused an expansion of the interval from 39.6 cM to 61.9 cM, were also removed. On the basis of consensus analysis of the six BC mapping populations, we obtained six corrected conflict-free variants of each chromosome, as well as an integrated map (based on all six datasets). Here, as an example, we provide a fragment of the integral map for chromosome 1 (Figure 6, dark and light ellipses represent shared and unique markers, respectively). This example demonstrates that consensus analysis resolves the conflict between the individual maps, *i.e.* brings the maps to one order of shared markers. Nevertheless, the obtained solution is not free of uncertainties, especially for unique markers (see *Discussion*).

#### Maize 24 RIL populations:

For each population, the data include marker scores for each of the 10 chromosomes. Unlike the previously mentioned BC data, these sets have a very high level of shared markers and a high quality of marker scoring. For each chromosome, we show the length of the map (in cM) based on ordering for each dataset separately (first row) and based on consensus analysis (second row). Except for a few cases (marked by frames in Table S4), the map lengths for individual-set ordering and consensus ordering were identical or very similar. This means that the unique maps built independently for each dataset displayed no conflicts of multilocus orders, pointing to a high-quality marker scoring. Still, for some set-chromosome combinations, the differences were moderate (3–10 cM) or rather high (>40 cM!). The last situation means that the cost of consensus is “not affordable” and that corresponding markers in the specific dataset should be removed, no matter which mechanism generated such a discrepancy of the specific population from all others. However, such a situation may also result from low marker density in some sets and real variation of marker positions among mapping populations, *e.g.* due to chromosomal rearrangements (see *Discussion*).

Here we provide more details on the above-noted case with considerable difference between the map length before and after consensus analysis (89.2 cM *vs.* 132.9 cM) for chromosome 9 in set 21. As in the BC data, one marker caused the effect. Here it was marker I00098. In this case, the effect was due to a very sparse map, which is actually represented by only four loci separated by 34, 28, and 36 cM. With such distances, unequivocal ordering is problematic (Figure S2). We can see from the illustration that unique order of marker I00098 in set 21 differs from the consensus order: the attempt for consensus resulted in moving this marker to the opposite end of the map, increasing the distance between I00098 and its closest neighbor from 36 cM to nearly 80 cM. It is noteworthy that, in all other sets, the size of the same interval varied from 7 cM in set 11 to 20 cM in set 22. This may mean either very high noise in scoring I00098 in set 21 or hotspot of recombination in this interval in the heterozygote from which set 21 was established.

For illustration, we show here the integral map of chromosome 1 (Figure S3). Obviously, this integral map differs significantly from the one shown for BC populations. This is due to the simple fact that BC data included a considerable proportion of non-shared markers, whereas in the RIL data, the proportion of such markers is close to zero. As a result, the integral map for the RIL problem is mainly represented as one-dimensional chain interrupted by some simple loops caused by unique patterns of chromosomal distribution of partially shared markers.

## Discussion

The application of the proposed approach based on synchronized-TSP formulation (hence, constrained discrete optimization) is illustrated on simulated data and real data on maize. In a comparison with other approaches, several aspects of the proposed methodology and employed criteria are worth discussing. Building multilocus maps can be based on various criteria, and choosing “the best” is not a simple task (Molinari *et al.* 2009).

**Figure 7:**
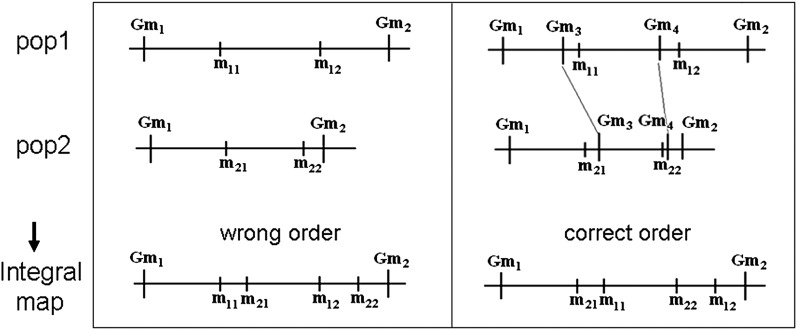
Correct positioning of unique (nonshared) markers on the integral map is not always possible if there are not enough shared markers. (Left) Wrong order of nonshared markers in the integral map built using the synthetic distance matrix constructed from two datasets, with unmeasured recombination rates calculated assuming proportional parallel changes of recombination along the chromosome (in our version of building the integral map, these markers form a loop). (Right) Correct order due to information provided by two additional shared markers in the considered chromosomal region.

Reduction of the consensus mapping problem to single-dataset mutilocus ordering using the synthetic distance matrix constructed from all datasets seems to be the simplest possible approach to the problem. It “automatically” resolves all conflicts because ordering is based on only one (synthetic) matrix. With this approach, irresolvable problems on mutual positioning of shared markers belonging to non-overlapping subsets are overlooked because of the assumption of proportional parallel changes of recombination rates along the chromosome in different sets that may contradict the reality (see example in Figure 7). In the considered example, *m_ij_* are population-specific (nonshared) markers. Marker *m*_22_ in population 2 is closer to the shared marker *Gm*_2_ than marker *m*_12_ in population 1, due to uneven and population-specific distribution of recombination along the segment. Without additional shared markers, this difference cannot be taken into account. Assuming “proportional” changes in recombination rates, one will get a wrong order, whereas the correct order will be obtained if additional shared markers *Gm*_3_ and *Gm*_4_ are available. Thus, with only two shared markers *Gm*_1_ and *Gm*_2_ in the region, the relative positions of markers *m_ij_* cannot be fully resolved, and employing surrogate information based on linear approximation of recombination rates will result in a wrong local order in the integral map. Similarly, a wrong order will be obtained for shared markers that belong to poorly overlapping subsets of mapping populations, if the missing experimental information on recombination rates between markers from these subsets is “derived” analytically based on linear approximation.

Much more reasonable is the graph-theoretic approach (GTA) based on the detection of non-conflicting subset of markers in the previously constructed individual maps (Yap *et al.* 2003; Jackson *et al.* 2008; Wu *et al.* 2008). There are some important advantages of the method presented in the current paper over GTA. In GTA, pairwise distances are employed between adjacent markers only, whereas our approach utilizes information from the whole distance matrix. Once conflicts among individual maps are detected, the correction in GTA is based on deleting some of the markers, whereas our approach is based on reordering the markers and utilizing the option of marker deletion only if the cost of consensus is high. If the initial maps were erroneous (due to small sample sizes, scoring errors, weak mapping algorithm, *etc*.), the GTA consensus map will absorb these errors (Jackson *et al.* 2008), whereas the approach proposed in this paper allows checking the data quality for each set based on resampling analysis, cleaning up the most problematic markers, and giving lower weight to datasets with inflated map lengths.

Choice of optimization criterion is the basis of any mapping approach. Here we extend the criterion of minimum total map length, widely employed by many authors dealing with multilocus map construction in the case of a single mapping population (*e.g.* Weeks and Lange 1987), to consensus mapping problem. For consensus mapping, we employ the sum of the map lengths taken across all datasets included in analysis. With this criterion, we are testing only those orders that fit the condition: shared order for shared markers. Obviously, this criterion can easily be extended to take into account the variation in information content in different datasets caused by variation in sample sizes and data quality. It is interesting that this approach may return correct multilocus order even in situations where intuitively clear and attractive voting criteria may result in wrong orders, *e.g.* with data different in signal/noise level (see example 2.2 above).

A specific aspect of the consensus mapping approach proposed here is the cost of consensus. This notion is closely related to the initial formulation of our approach, with synchronous re-analysis of the initial mapping data based on testing only non-conflicting multilocus orders. Consensus ordering will be accompanied with high cost when some of the markers in the initial maps (constructed during separate analysis of each population) appear in conflict. In fact, consensus analysis moves such markers to new positions, resulting in map expansion compared with the initial map. Local corrections of marker positions during consensus analysis will cause insignificant increase in map length, but considerable changes cannot be excluded, as could be seen from our examples on real data. There might be several reasons for such considerable changes, calling for careful analysis followed by corresponding decision making (*e.g.* deleting the problematic marker from one or several datasets). Indeed, one of the reasons of high cost may be high noise in marker scoring in one of the populations. But biological reasons can also play a role, including chromosomal rearrangements (*e.g.* Nelson *et al.* 1995). In such cases, a marker may have a different map position in a certain population compared with other populations (in principle, chromosomal rearrangement may also result in more serious changes of map order, involving considerable segments). With the graph-theoretic approach, the previously mentioned problem is absent because the trouble-making markers are always deleted. With our approach, the need to remove a marker due to high cost of consensus is quite rare. Usually, local repositioning of the markers resolves the noise-generated conflicts without substantial increase in map length.

Although the general scheme of our approach includes synchronous optimization over the entire chromosomal length (global analysis), a higher computational efficiency was achieved by local analysis based on segmentation of the total map into regions of local conflicts separated by regions of no conflicts and employing the consensus optimization criterion for each conflict region separately. What are the reasons justifying the use of local analysis? The main cause of conflicts is the noise in recombination data, originating from three factors: errors in marker allele identification; missing data for some or many markers; and statistical noise decreasing with sample size. Low noise does not affect the quality of multilocus ordering (Mester *et al.* 2003a). With increasing noise, local wrong orders will be manifested as local conflicts between the individual maps. Further increase in the noise level will cause a widening of the conflict regions that will still be local, and only rare moderate noise will throw a marker far enough. Thus, local analysis is justified by the assumption of low-to-moderate noise in recombination scores. The other assumption is that resolving local conflicts should provide the same solution as global analysis based on optimization of the main criterion (total map length, equation 2). Obviously, larger size of the entire chromosome compared with the sizes of regions of local conflicts considerably complicates the global solution process using heuristic algorithms.

Unlike many applications of TSP, where differences in portions of percentage in the achieved optimal value of the criterion have no practical value, in genetic mapping, the value of the criterion is not important by itself. However, even small differences in the criterion value can be associated with considerable deviation of the calculated order from the true order. Therefore, as the first step in the consensus analysis, we conduct ordering separately for each dataset taking advantage of jackknife resampling verification of the obtained orders (Mester *et al.* 2003a). The next step is detection of conflicts and local analysis to resolve the conflicts keeping the non-conflict regions “protected” (frozen). With small sizes of the conflict region, an exact analysis is affordable; otherwise, we employ heuristic algorithms for synchronous-TSP optimization (Figure 4). At this step, the cost of consensus should be taken into account to prevent considerable map length inflation due to noisy markers. After all local conflicts are resolved, the obtained solution can be employed as a starting point to run the global optimization over the entire chromosome to ensure that the obtained solution cannot be further improved.

An interesting perspective for improving the quality of consensus analysis may be mutual controlling of the results obtained with synchronous-TSP heuristics and GTA. Namely, the multilocus orders constructed by reanalysis or raw data and coordinated with resampling quality control may be employed as a starting point for both approaches. Although GTA is less sparing (less economical) with respect to conflicting markers (hence, fewer markers will remain in the GTA consensus maps than in the synchronous-TSP maps), if the two approaches perform well, the subset of markers remaining in the final consensus maps from the two methods are expected to be in the same order. Such correspondence will point to the quality of the solution. Other possibilities of building hybrid algorithms taking advantage of the complementary properties of the two approaches remain to be investigated.

## Supplementary Material

Supporting Information
