# Supplementary material for: Two-Phase Analysis in Consensus Genetic Mapping
Source: G3 (Bethesda). 2012 May 1;2(5):537–49. doi: 10.1534/g3.112.002428 (PMC3362937; doi:10.1534/g3.112.002428)
Supplement: Supporting Information [file supp_2_5_537__index.html]

Supporting Information 

# Two-Phase Analysis in Consensus Genetic Mapping

## Supporting Information for Ronin *et al*, 2012

**Files in this Data Supplement:**

- Supporting Information - Figures S1-S3, Files S1-S3, Tables S1-S4 (PDF, 645 KB)
- Figure S1 - Consensus orders containing markers *m25-m26* (PDF, 74 KB)
- Figure S2 - A rare example when high cost of consensus cannot be reduced by deleting some marker(s) (PDF, 92 KB)
- Figure S3 - The integral map of chromosome 1 (based on data of 24 maize RIL populations of maize) (PDF, 361 KB)
- Table S1 - Effect of markers scoring errors (se) on accuracy of multilocus ordering in individual and consensus mapping (simulated data of Example 2.1) (PDF, 70 KB)
- Table S2 - The advantage of the weighted criterion of sum of recombination rates over the voting criterion in the presence of both pure and very noisy data (simulated data of Example 2.2) (PDF, 63 KB)
- Table S3 - The lengths of the chromosomes after individual and consensus analysis allowing to detect the chromosomes with high cost of consensus ordering: data on six BC maize sets (PDF, 68 KB)
- Table S4 - The lengths of the chromosomes after individual and consensus analysis allowing to detect the chromosomes with high cost of consensus ordering: data on 24 RIL maize sets (PDF, 106 KB)
- File S1 - This file includes single-chromosome data on 16 F2 populations each with sample size 150 genotypes scored for 50 codominant markers (.zip, 50 KB)
- File S2 - In contrast to the previous example, no missing data were in the first 8 populations but their sample size was only 50 per population, i.e., 1/3 of the sample size of the second group of 8 populations (.zip, 31 KB)
- File S3 - Here the sample size for each of the 16 populations was 100 (.zip, 27 KB)
