# Supplementary material for: Two-Phase Analysis in Consensus Genetic Mapping
Source: G3 (Bethesda). 2012 May 1;2(5):537–49. doi: 10.1534/g3.112.002428 (PMC3362937; doi:10.1534/g3.112.002428)
Supplement: Supporting Information [file supp_2.5.537_002428SI.pdf]

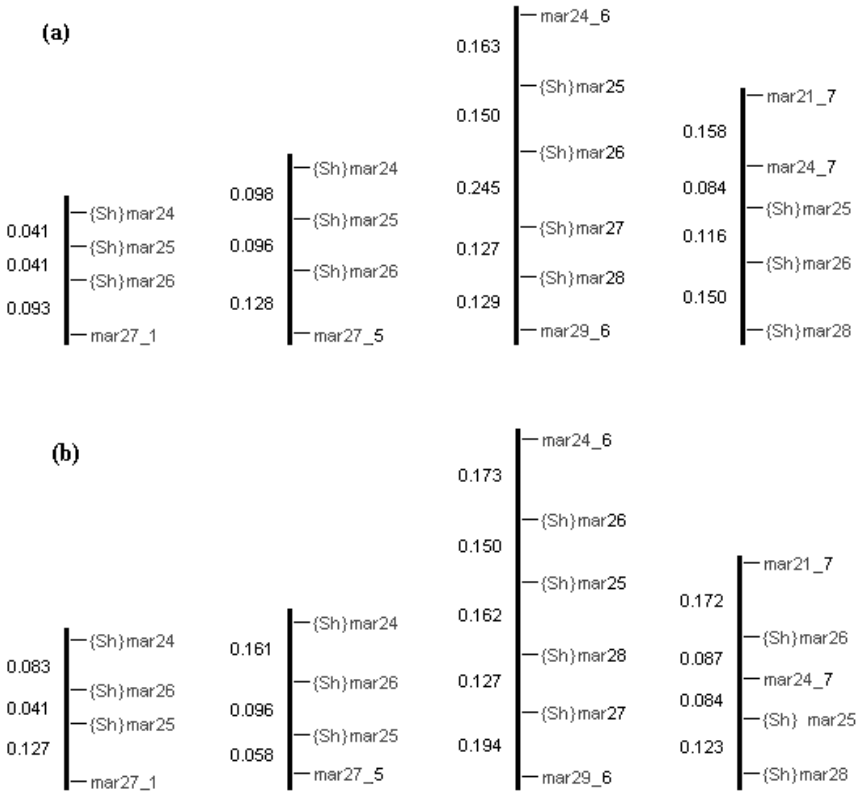

Figure S1 Consensus orders containing markers *m25-m26*: (a) request of consensus elongates maps of set 1 from the first group (without missing data and scoring errors); (b) request of consensus elongates maps of sets 2, 3, and 4 from the second group (with missing data and scoring errors). In total, the sum of lengths 1.82 for correct (simulated) order was smaller than that for the incorrect order equal to 1.84.

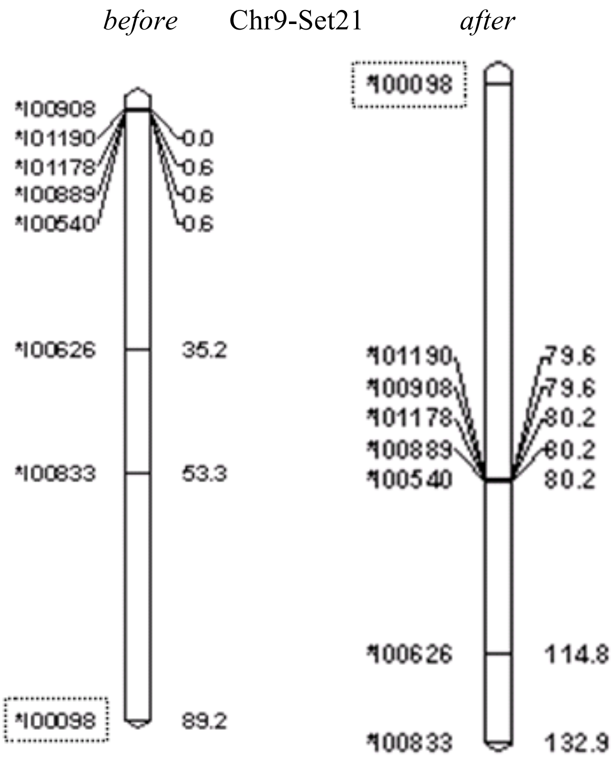

Figure S2 A rare example when high cost of consensus cannot be reduced by deleting some marker(s).

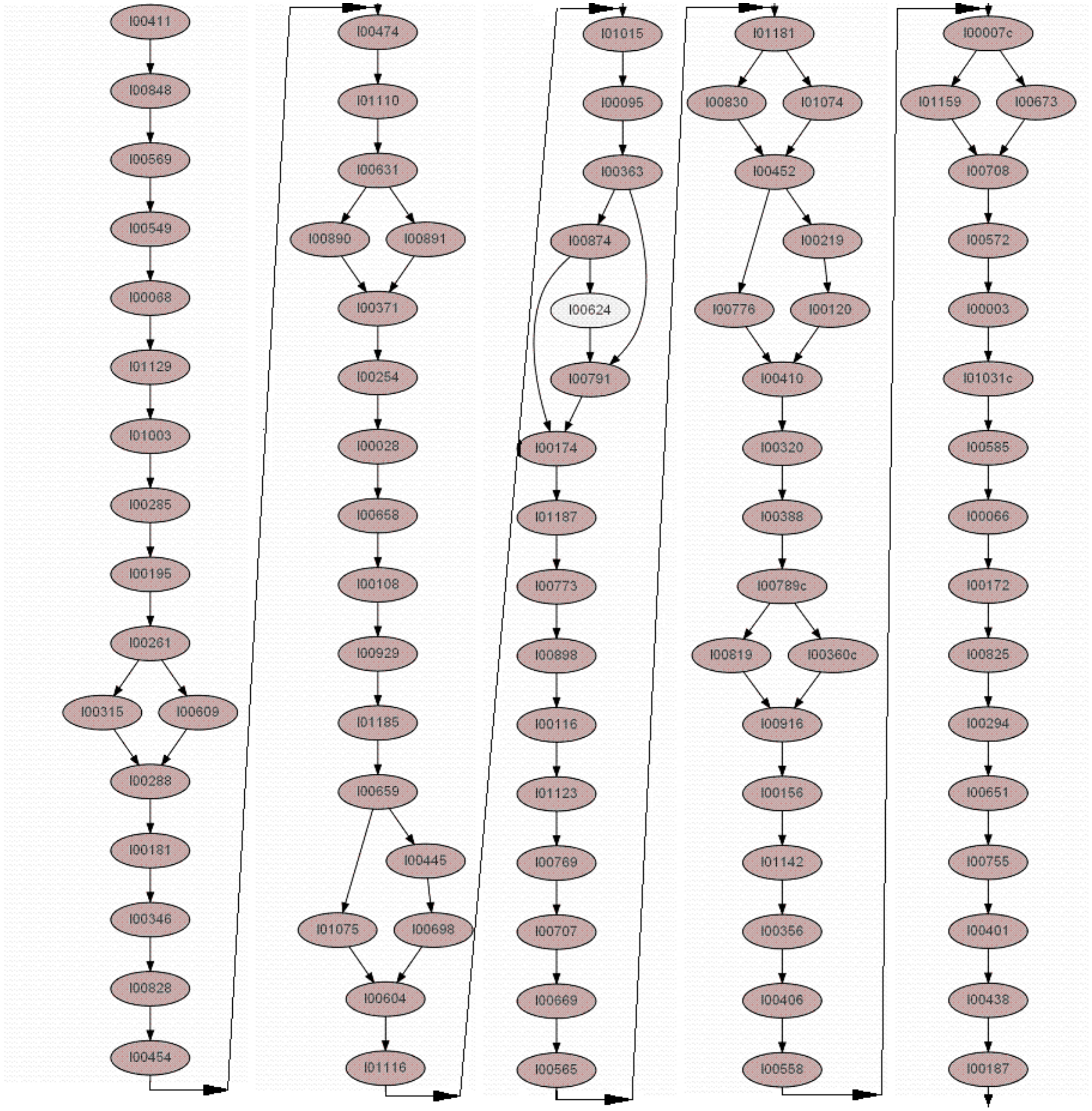
I00411
I00848
I00569
I00549
I00068
I01129
I01003
I00285
I00195
I00261
I00315
I00609
I00288
I00181
I00346
I00828
I00454
I00474
I01110
I00631
I00890
I00891
I00371
I00254
I00028
I00658
I00108
I00929
I01185
I00659
I00445
I01075
I00698
I00604
I01116
I01015
I00095
I00363
I00874
I00624
I00791
I00174
I01187
I00773
I00898
I00116
I01123
I00769
I00707
I00669
I00565
I01181
I00830
I01074
I00452
I00219
I00776
I00120
I00410
I00320
I00388
I00789c
I00819
I00360c
I00916
I00156
I01142
I00356
I00406
I00558
I00007c
I01159
I00673
I00708
I00572
I00003
I01031c
I00585
I00066
I00172
I00825
I00294
I00651
I00755
I00401
I00438
I00187

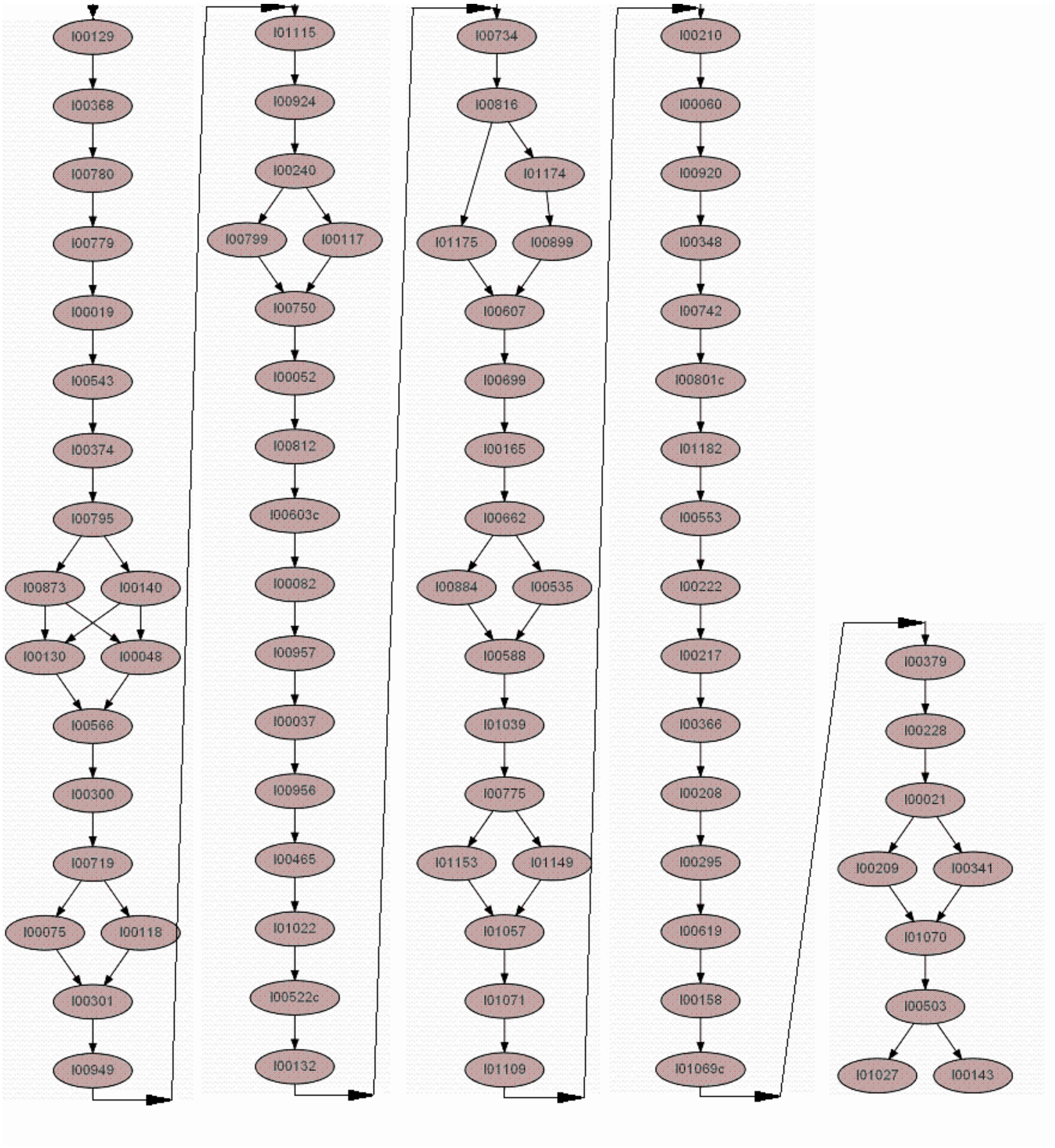

Figure S3 The integral map of chromosome 1 (based on data of 24 maize RIL populations of maize). The predominantly linear mode of the map (relatively rare regions of branching) is caused by high proportion of shared markers and high overlap of the sets for the marker content.

Files S1-S3
Supporting Data

Files S1-S3 are available for download at http://www.g3journal.org/lookup/suppl/doi:10.1534/g3.112.002428/-/DC1.

File S1 – This file includes single-chromosome data on 16 F_2 populations each with sample size 150 genotypes scored for 50 codominant markers.

File S2 - In contrast to the previous example, no missing data were in the first 8 populations but their sample size was only 50 per population, i.e., 1/3 of the sample size of the second group of 8 populations.

File S3 - Here the sample size for each of the 16 populations was 100. The first 8 populations were simulated with no missing data, while 20% of missing data was simulated for the remaining 8 populations.

Table S1 Effect of markers scoring errors (se) on accuracy of multilocus ordering in individual and consensus mapping (simulated data of Example 2.1)

Set #	1	2	3	4	5	6	7	8	9	10	11	12	13	14	15	16
se, %	←			0				→	←			10				→
Ind(cM)	244	182	251	287	311	330	253	313	552	578	542	462	528	568	535	574
Cons(cM)	249	182	251	287	311	330	253	313	567	589	554	467	542	576	545	584
	1	1	3	4	3	8	1	3	**4** 1	1	**4** 3	**4** 1	**2** 1	3	2	3
	4	6	5	5	5	9	2	5	**1** 2	**3** 2	**3** 4	**1** 4	**3** 2	**11** 10	4	8
	5	7	6	8	7	14	3	8	**2** 3	**2** 3	7	6	**1** 3	**10** 11	5	10
	8	8	7	10	8	15	7	9	**3** 4	4	8	8	5	13	7	11
	9	9	10	11	13	16	10	13	7	6	9	9	7	14	8	18
	10	11	15	12	18	17	11	18	10	**13** 11	11	13	8	16	12	21
	11	13	20	13	20	21	13	20	11	**11** 13	13	**19** 17	13	17	**14** 13	25
	12	14	21	20	21	27	16	21	12	22	15	**17** 19	**19** 19	22	**16** 14	26
	15	16	23	21	22	32	19	24	13	25	**17** 16	26	**18** 18	24	**13** 16	29
	16	19	25	24	23	33	23	26	14	**28** 27	**16** 17	27	21	25	17	30
	18 **19**	21	27	27	28	38	25	27	17	**27** 28	28	28	**25** 24	26	19	31
	19 **18**	24	30	28	29	40	26	28	18	30	29	30	**24** 25	30	20	**35** 33
	24	26	34	31	38	41	28	29	21	32	30	32	**27** 26	35	**26** 22	**33** 34
	25	27	35	32	39	45	29	30	23	33	31	33	**26** 27	36	**22** 26	**36** 35
	29	28	36	34	43	47	30	32	24	34	33	34	31	38	27	**34** 36
	31	30	37	35	45	49	31	33	**26** 25	36	34	**36** 35	32	40	**30** 28	38
	34	34	38	36	47	50	34	34	**25** 26	**38** 37	36	**37** 36	**34** 33	42	**28** 30	39
	35	38	41	38	50		36	36	28	**37** 38	39	**35** 37	**33** 34	46	31	40
	37	39	42	41			37	37	29	**43** 40	40	39	**36** 35	49	**33** 32	**46** 42
	39	42	43	42			40	39	**32** 31	**40** 41	**45** 42	40	**37** 36		**32** 33	**44** 43
	41	43	45	46			45	40	**31** 32	**41** 42	**46** 44	**46** 44	**35** 37		38	**42** 44
	42	45	46	50			42	41	33	**42** 43	**44** 45	**44** 45	38		40	**43** 46
	43	47	47				44	45	35	45	**42** 46	**45** 46	39		43	48
	45	50	48				47	46	**41** 40	47	47	49	40		44	50

46	50	**40** 41	50	50	41	**46** 45
47		45			**43** 42	**45** 46
50		47			**42** 43	47
		49			45	48
		50			**49** 48	49
					**48** 49	50

Table S2 The advantage of the weighted criterion of sum of recombination rates over the voting criterion in the presence of both pure and very noisy data (simulated data of Example 2.2).

Set #	1	2	3	4	5		6		7		8	
se %	←	0		→	←		10		→			
Ind(cM)	244	182	251	287	552		578		542		462	
Cons(cM)	244	182	251	287	567		590		556		468	
	1	1	3	4	**4**	1	1		**4**	3	**4**	1
	4	6	5	5	**1**	2	**3**	2	**3**	4	**1**	4
	5	7	6	8	**2**	3	**2**	3	7		6	
	8	8	7	10	**3**	4	4		8		8	
	9	9	10	11	7		6		9		9	
	10	11	15	12	10		**13**	11	11		13	
	11	13	20	13	11		**11**	13	13		**19**	17
	12	14	21	20	12		**26**	25	15		**17**	19
	15	16	23	21	13		**25**	26	**17**	17	26	
	16	19	25	24	14		**28**	27	**16**	16	27	
	19	21	27	27	17		**27**	28	**26**	25	28	
	24	24	30	28	21		30		**25**	26	30	
	25	26	34	31	23		32		28		32	
	26	27	35	32	24		33		29		33	
	29	28	36	34	**26**	25	34		30		34	
	31	30	37	35	**25**	26	36		31		**36**	35
	34	34	38	36	28		**38**	37	33		**37**	36
	35	38	41	38	29		**37**	38	34		**35**	37
	37	39	42	41	**32**	31	**43**	40	36		39	
	39	42	43	42	**31**	32	**40**	41	39		40	
	41	43	45	46	33		**41**	42	40		**46**	44
	42	45	46	50	35		**42**	43	**45**	44	**44**	45
	43	47	47		**41**	40	45		**46**	42	**45**	46
	45	50			**40**	41	47		**44**	45	49	
	46				45		50		**42**	46	50	
	47				47				47			
	50				49							
					50							

Table S3 The lengths of the chromosomes after individual and consensus analysis allowing to detect the chromosomes with high cost of consensus ordering: data on six BC maize sets

Chr #	Phase	set1	set2	set3	set4	set5	set6
1	*before*	243.6	212.0	226.8	184.9	202.5	234.2
	after	245.7	212.1	226.8	184.9	203.6	234.2
2	*before*	146.5	144.8	121.2	60.5	170.3	95.5
	after	146.5	167.6	123.4	61.5	170.4	95.5
	after-crt	146.5	144.8	123.4	61.5	170.3	95.5
3	*before*	154.1	167.8	147.9	172.8	140.3	117.0
	after	154.1	167.8	147.9	172.8	144.6	117.0
4	*before*	100.6	158.9	142.0	144.4	123.9	105.2
	after	100.6	158.7	142.0	144.4	123.9	105.2
5	*before*	143.5	157.5	158.3	162.3	136.2	151.2
	before-crt	143.5	157.5	135.2	162.3	136.2	151.2
	after	143.5	157.5	166.4	163.5	136.2	151.2
	after-crt	143.5	157.5	137.8	163.5	136.2	151.2
6	*before*	104.5	139.6	53.6	120.9	125.6	83.4
	after	104.5	139.8	53.6	211.8	128.9	83.4
	after-crt	104.5	139.7	53.6	120.7	125.7	83.4
7	*before*	111.9	139.8	191.7	111.8	124.3	92.0
	before-crt	111.9	139.8	162.8	111.8	124.3	92.0
	after	111.9	139.8	200.6	111.8	124.3	92.0
	after-crt	111.9	139.8	167.2	111.8	124.3	92.0
8	*before*	133.4	141.8	114.5	104.6	145.7	86.7
	after	133.4	141.8	121.0	104.6	145.7	86.7
9	*before*	132.3	119.1	136.3	139.8	97.8	46.2
	after	132.3	119.1	136.3	139.8	97.8	46.2
10	*before*	97.2	114.1	112.1	75.9	109.3	58.9
	after	97.2	114.2	115.3	75.9	111.5	58.9

Notes: *before* – the lengths (in cM) of the map variants after individual analysis of separate sets but before the consensus analysis; *before-crt* – the same as *before* but after removing some markers of chromosomes 5 and 7 in set #3; *after* - the legths of the map variants after the consensus analysis; *after-crt* – the same as *after* but after removing some markers of set2-Chr2, set3-Chr5, set4-Chr6, and set3-Chr7

Table S4 The lengths of the chromosomes after individual and consensus analysis allowing to detect the chromosomes with high cost of consensus ordering: data on 24 RIL maize sets

Chr	b/a	Sets (mapping populations)											
Chr 1		set1	set2	set3	set4	set5	set6	set7	set8	set9	set10	set11	set12
	b	170.5	158.1	198.3	160.8	201.2	175.2	151.8	180.7	166.8	164.1	160.2	157.2
	a	170.6	159.6	199.7	161.5	203.3	175.6	153.4	182.0	169.0	164.9	161.2	159.8
		set14	set15	set16	set18	set19	set20	set21	set22	set23	set24*	set25*	set26
	b	166.8	143.1	157.2	149.8	172.2	188.8	131.0	165.1	113.1	134.4	139.1	179.3
	a	170.1	143.1	157.3	150.3	172.8	189.4	131.0	165.8	113.9	134.8	139.5	179.6
Chr 2		set1	set2	set3	set4	set5	set6	set7	set8	set9	set10	set11	set12
	b	131.5	120.9	161.1	124.5	133.4	150.2	134.0	143.3	123.3	136.2	123.1	148.1
	a	132.0	122.1	161.4	126.1	134.1	150.2	134.4	143.8	124.0	137.1	123.1	148.8
		set14	set15	set16*	set18	set19	set20	set21	set22	set23*	set24*	set25	set26*
	b	112.1	103.3	43.3	116.3	121.8	138.4	64.9	131.2	44.5	39.1	145.8	35.0
	a	112.4	103.3	43.5	116.7	121.8	138.7	64.9	132.0	44.9	39.2	145.8	35.0
Chr 3		set1*	set2	set3	set4	set5	set6	set7	set8	set9	set10	set11	set12
	b	77.1	127.8	137.4	138.5	32.9	97.2	118.0	144.8	120.1	145.6	140.1	143.5
	a	77.1	128.9	138.3	138.7	34.0	97.6	118.0	144.8	120.3	146.9	140.3	143.8
		set14	set15	set16	set18	set19	set20	set21	set22	set23	set24	set25	set26
	b	125.0	-	117.5	107.2	114.8	137.8	135.7	113.1	-	54.4	98.3	80.0
	a	125.1	-	118.4	107.2	115.2	138.1	135.7	113.1	-	54.9	98.3	80.0
Chr 4		set1	set2	set3	set4	set5*	set6	set7	set8	set9	set10	set11	set12
	b	121.3	121.6	132.9	99.0	59.8	65.4	97.4	109.7	111.4	112.0	77.4	72.0
	a	121.6	121.9	133.2	99.6	60.4	65.4	97.9	109.8	112.0	112.0	77.4	72.0
		set14	set15	set16	set18	set19	set20	set21	set22	set23	set24	set25	set26
	b	93.1	49.2	103.4	103.1	113.5	114.9	51.5	125.8	19.5	-	130.7	97.3
	a	93.1	49.2	103.4	103.1	113.5	114.9	51.8	126.1	19.6	-	131.3	100.7
Chr 5		set1	set2	set3	set4	set5	set6	set7	set8	set9	set10	set11	set12
	b	116.	101.	134.6	128.	149.8	147.	122.	143.8	136.4	129.2	119.0	106.5

	2	4		9		4	6					
a	116.9	101.5	137.0	130.1	150.4	148.1	123.2	145.0	136.6	129.5	119.1	107.4
	set14	set15	set16	set18	set19*	set20	set21	set22*	set23	set24	set25	set26
b	113.1	-	104.0	121.6	77.8	104.2	91.2	65.3	-	91.2	96.5	67.5
a	115.8	-	104.9	122.8	78.4	106.3	91.9	66.2	-	91.2	97.1	67.6

Table 4s continued

Chr6		set1	set2	set3	set4	set5	set6	set7*	set8	set9	set10	set11	set12
	b	77.4	92.5	95.2	79.7	96.1	95.0	39.0	101.1	50.1	88.7	83.8	90.5
	a	77.4	93.6	99.0	79.9	96.2	95.0	39.0	101.4	51.0	88.7	83.8	90.6
		set14	set15	set16	set18	set19	set20*	set21	set22	set23	set24	set25	set26
	b	85.9	100.0	82.4	90.6	86.1	38.8	41.8	87.9	15.4	58.2	101.3	107.1
	a	86.2	100.8	82.4	90.8	86.5	38.8	42.4	88.2	15.4	58.2	101.3	107.1
Chr7		set1	set2	set3*	set4*	set5	set6	set7	set8	set9	set10	set11	set12
	b	106.5	103.8	58.8	45.1	65.9	101.7	87.2	108.4	118.8	110.0	105.7	109.3
	a	106.9	103.8	59.2	45.1	65.9	101.7	87.2	112.8	118.8	110.2	105.9	109.6
		set14	set15*	set16	set18	set19	set20	set21	set22	set23	set24	set25	set26
	b	114.6	49.3	107.9	81.9	108.9	68.0	71.4	110.5	49.4	0.3	114.5	105.6
	a	115.3	49.3	108.3	82.2	109.8	68.0	71.7	117.4	49.5	0.3	114.5	107.6
Chr8		set1	set2	set3	set4	set5	set6	set7	set8	set9	set10	set11	set12
	b	107.1	113.3	121.0	91.2	101.3	110.9	69.0	115.7	117.5	116.4	98.4	97.1
	a	107.4	113.6	121.7	92.0	101.6	110.9	69.0	116.1	117.6	116.4	98.8	97.1
		set14	set15	set16	set18	set19	set20	set21	set22	set23	set24	set25	set26
	b	93.5	79.2	105.7	99.1	101.9	96.8	117.5	117.2	62.0	65.4	68.7	116.0
	a	94.1	79.3	106.0	99.1	102.3	96.8	117.5	117.5	62.0	65.4	68.7	116.8
Chr9		set1	set2	set3	set4	set5	set6	set7	set8	set9	set10	set11	set12
	b	89.4	93.2	105.5	97.8	65.9	76.9	91.1	104.5	93.7	105.7	110.0	94.6
	a	89.9	94.1	110.8	98.5	65.9	77.3	91.7	104.5	94.1	106.3	110.9	94.6
		set14	set15	set16*	set18	set19	set20	set21	set22	set23	set24	set25	set26
	b	85.4	80.4	59.7	90.6	92.2	76.5	89.2	94.5	48.4	15.5	106.7	106.5
	a	85.5	81.2	62.6	92.0	92.5	76.6	132.9	94.5	50.2	15.5	106.7	106.9
Chr10		set1	set2	set3	set4	set5	set6	set7	set8	set9	set10	set11	set12
	b	71.6	92.8	98.3	89.8	-	74.2	77.2	81.4	82.9	90.4	91.0	80.1
	a	71.6	93.6	98.3	90.5	-	74.2	77.7	81.4	82.9	90.4	91.0	80.1
		set14*	set15	set16	set18	set19	set20	set21	set22	set23	set24	set25	set26*
	b	53.1	81.8	80.7	73.2	86.8	103.1	75.8	84.5	34.6	58.1	88.6	6.4
	a	53.1	81.8	81.0	73.6	86.8	103.1	75.8	84.8	34.6	58.1	89.0	6.4

Notes: b and a – represent two phases, ***b**efore* and ***a**fter* of the proposed consensus mapping approach. Set-chromosome combinations with high or moderate cost of consensus are marked by frames (especially set21-Chr9). The situation with set21-Chr9 is discussed in more detail in the text. * marks the sets where the considered chromosome is represented by several fragment (linkage groups); for such cases the sum of the fragment lengths (in cM) is presented.
