# Supplementary material for: Two-Phase Analysis in Consensus Genetic Mapping
Source: G3 (Bethesda). 2012 May 1;2(5):537–49. doi: 10.1534/g3.112.002428 (PMC3362937; doi:10.1534/g3.112.002428)
Supplement: Supporting Information [file supp_2.5.537_FigureS1.pdf]

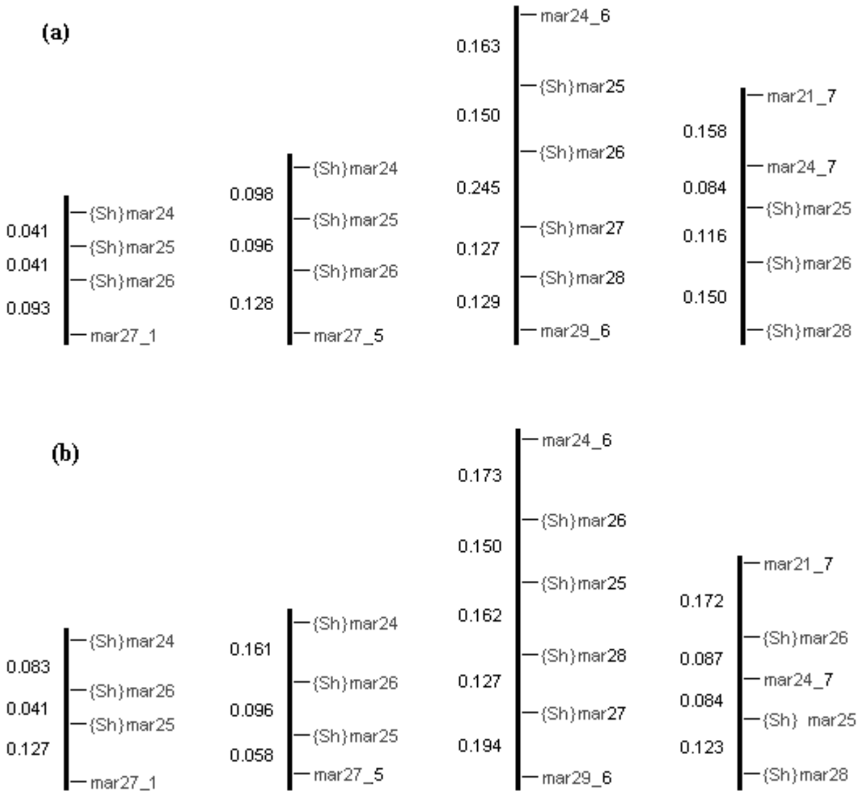

Figure S1 Consensus orders containing markers *m25-m26*: (a) request of consensus elongates maps of set 1 from the first group (without missing data and scoring errors); (b) request of consensus elongates maps of sets 2, 3, and 4 from the second group (with missing data and scoring errors). In total, the sum of lengths 1.82 for correct (simulated) order was smaller than that for the incorrect order equal to 1.84.
