# Supplementary figures and images for: Two-Phase Analysis in Consensus Genetic Mapping
Source: G3 (Bethesda). 2012 May 1;2(5):537–49. doi: 10.1534/g3.112.002428 (PMC3362937; doi:10.1534/g3.112.002428)

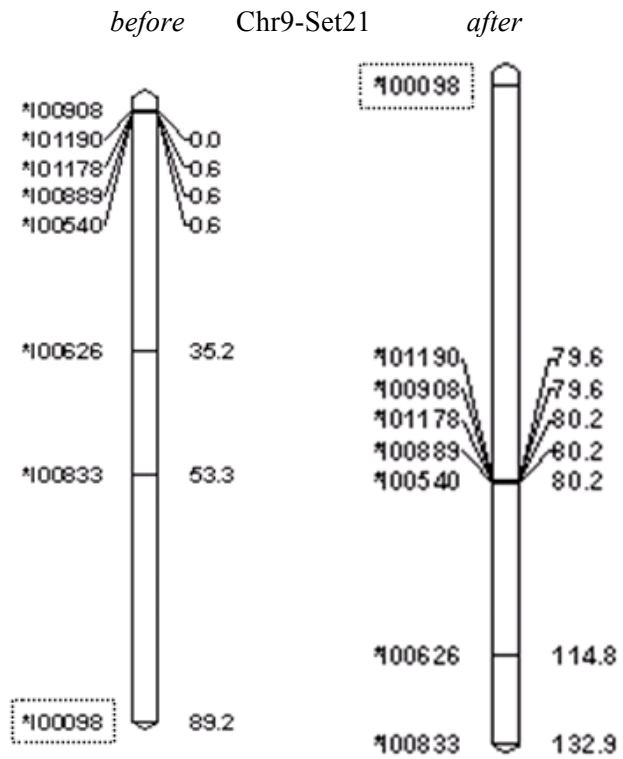

**Figure S2** A rare example when high cost of consensus cannot be reduced by deleting some marker(s).

Supplement: Supporting Information [file supp_2.5.537_FigureS2.pdf]
