# Supplementary material for: Two-Phase Analysis in Consensus Genetic Mapping
Source: G3 (Bethesda). 2012 May 1;2(5):537–49. doi: 10.1534/g3.112.002428 (PMC3362937; doi:10.1534/g3.112.002428)
Supplement: Supporting Information [file supp_2.5.537_FigureS3.pdf]

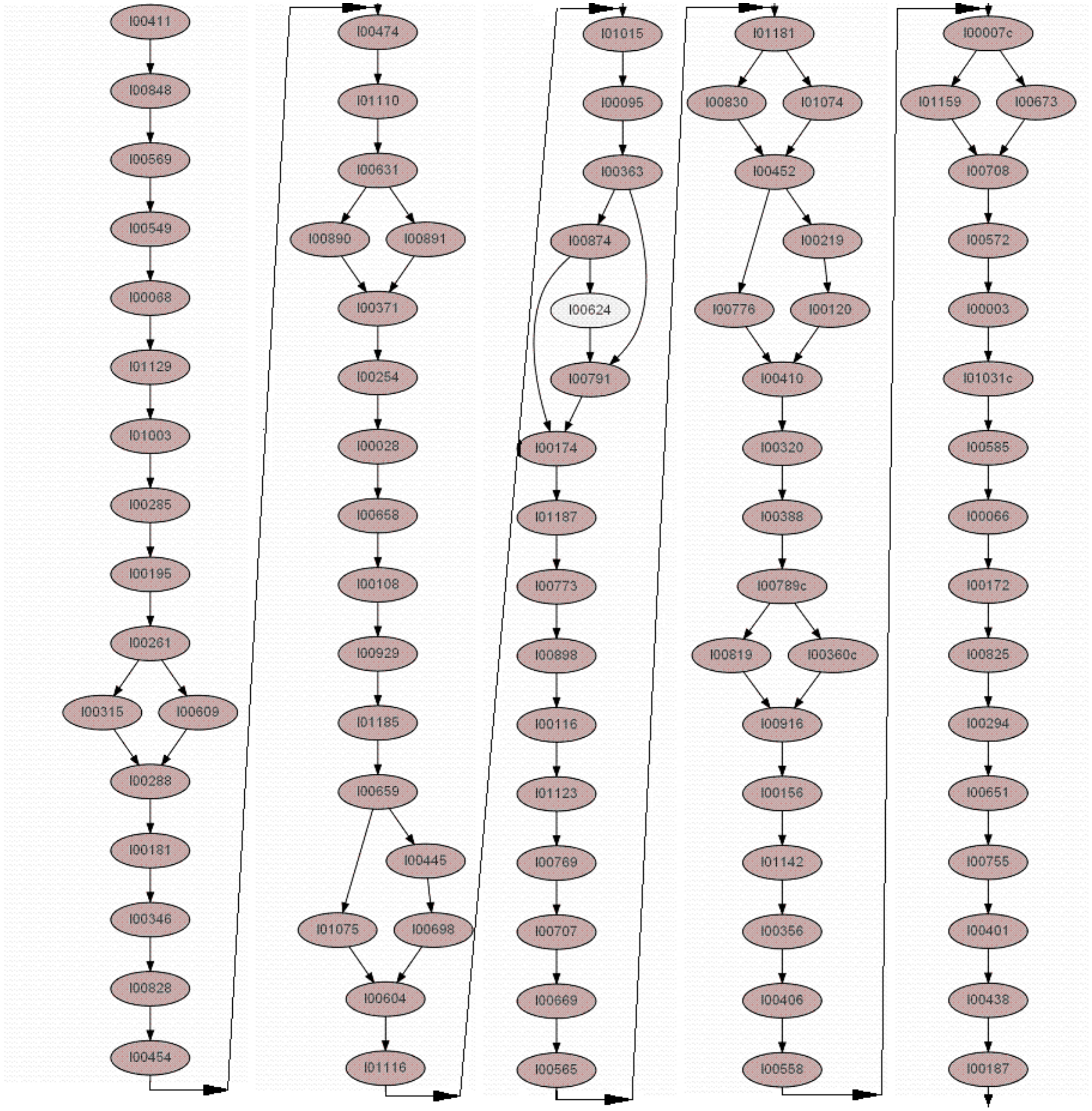
I00411
I00848
I00569
I00549
I00068
I01129
I01003
I00285
I00195
I00261
I00315
I00609
I00288
I00181
I00346
I00828
I00454
I00474
I01110
I00631
I00890
I00891
I00371
I00254
I00028
I00658
I00108
I00929
I01185
I00659
I00445
I01075
I00698
I00604
I01116
I01015
I00095
I00363
I00874
I00624
I00791
I00174
I01187
I00773
I00898
I00116
I01123
I00769
I00707
I00669
I00565
I01181
I00830
I01074
I00452
I00219
I00776
I00120
I00410
I00320
I00388
I00789c
I00819
I00360c
I00916
I00156
I01142
I00356
I00406
I00558
I00007c
I01159
I00673
I00708
I00572
I00003
I01031c
I00585
I00066
I00172
I00825
I00294
I00651
I00755
I00401
I00438
I00187

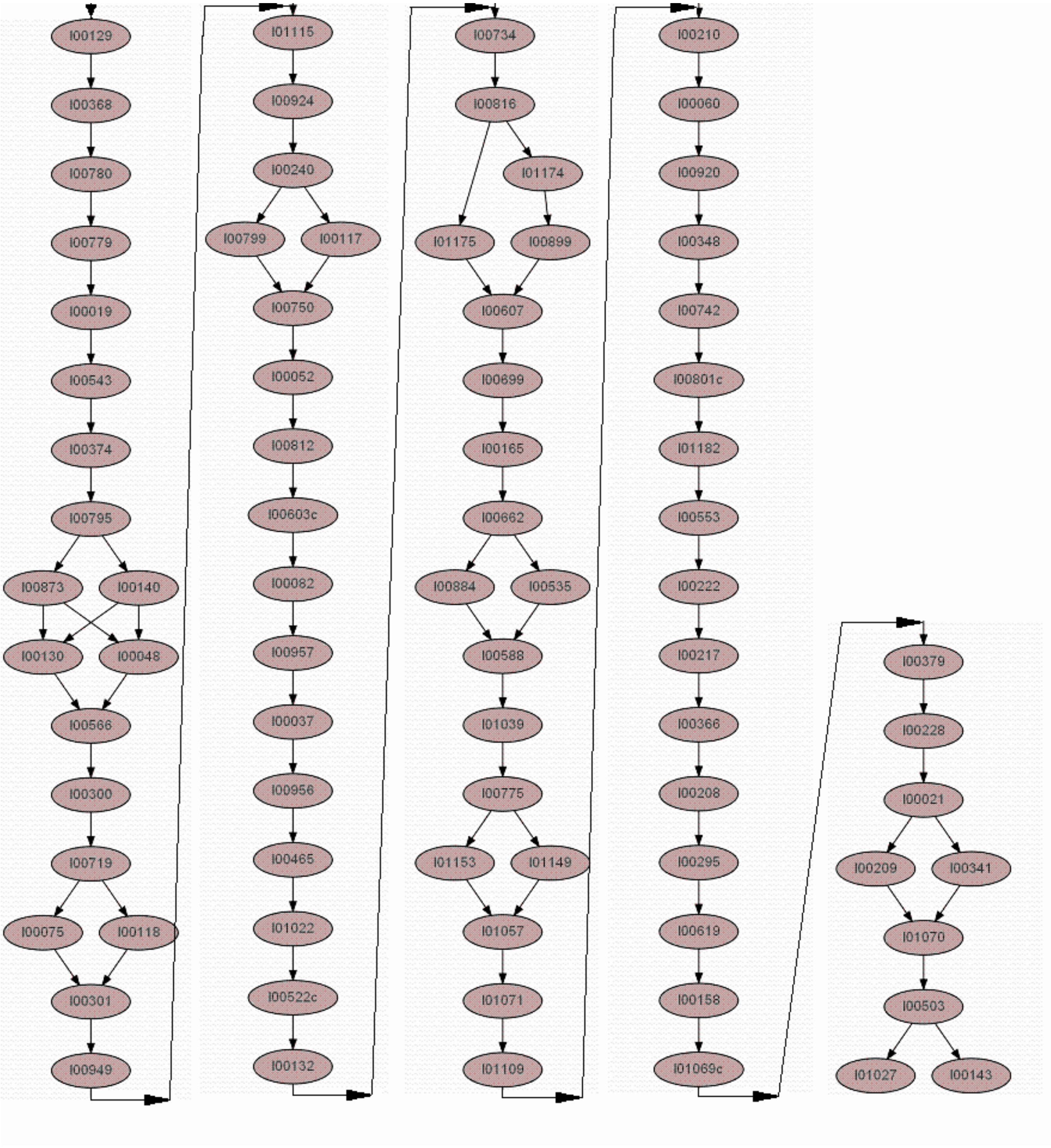

Figure S3 The integral map of chromosome 1 (based on data of 24 maize RIL populations of maize). The predominantly linear mode of the map (relatively rare regions of branching) is caused by high proportion of shared markers and high overlap of the sets for the marker content.
