# Supplementary material for: Two-Phase Analysis in Consensus Genetic Mapping
Source: G3 (Bethesda). 2012 May 1;2(5):537–49. doi: 10.1534/g3.112.002428 (PMC3362937; doi:10.1534/g3.112.002428)
Supplement: Supporting Information [file supp_2.5.537_TableS1.pdf]

**Table S1 Effect of markers scoring errors (se) on accuracy of multilocus ordering in individual and consensus mapping (simulated data of Example 2.1)**

| Set # | 1 | 2 | 3 | 4 | 5 | 6 | 7 | 8 | 9 | 10 | 11 | 12 | 13 | 14 | 15 | 16 |
|---|---|---|---|---|---|---|---|---|---|---|---|---|---|---|---|---|
| se, % | ← | | | 0 | | | | → | ← | | | 10 | | | | → |
| Ind(cM) | 244 | 182 | 251 | 287 | 311 | 330 | 253 | 313 | 552 | 578 | 542 | 462 | 528 | 568 | 535 | 574 |
| Cons(cM) | 249 | 182 | 251 | 287 | 311 | 330 | 253 | 313 | 567 | 589 | 554 | 467 | 542 | 576 | 545 | 584 |
| | 1 | 1 | 3 | 4 | 3 | 8 | 1 | 3 | **4** 1 | 1 | **4** 3 | **4** 1 | **2** 1 | 3 | 2 | 3 |
| | 4 | 6 | 5 | 5 | 5 | 9 | 2 | 5 | **1** 2 | **3** 2 | **3** 4 | **1** 4 | **3** 2 | **11** 10 | 4 | 8 |
| | 5 | 7 | 6 | 8 | 7 | 14 | 3 | 8 | **2** 3 | **2** 3 | 7 | 6 | **1** 3 | **10** 11 | 5 | 10 |
| | 8 | 8 | 7 | 10 | 8 | 15 | 7 | 9 | **3** 4 | 4 | 8 | 8 | 5 | 13 | 7 | 11 |
| | 9 | 9 | 10 | 11 | 13 | 16 | 10 | 13 | 7 | 6 | 9 | 9 | 7 | 14 | 8 | 18 |
| | 10 | 11 | 15 | 12 | 18 | 17 | 11 | 18 | 10 | **13** 11 | 11 | 13 | 8 | 16 | 12 | 21 |
| | 11 | 13 | 20 | 13 | 20 | 21 | 13 | 20 | 11 | **11** 13 | 13 | **19** 17 | 13 | 17 | **14** 13 | 25 |
| | 12 | 14 | 21 | 20 | 21 | 27 | 16 | 21 | 12 | 22 | 15 | **17** 19 | **19** 19 | 22 | **16** 14 | 26 |
| | 15 | 16 | 23 | 21 | 22 | 32 | 19 | 24 | 13 | 25 | **17** 16 | 26 | **18** 18 | 24 | **13** 16 | 29 |
| | 16 | 19 | 25 | 24 | 23 | 33 | 23 | 26 | 14 | **28** 27 | **16** 17 | 27 | 21 | 25 | 17 | 30 |
| | 18 **19** | 21 | 27 | 27 | 28 | 38 | 25 | 27 | 17 | **27** 28 | 28 | 28 | **25** 24 | 26 | 19 | 31 |
| | 19 **18** | 24 | 30 | 28 | 29 | 40 | 26 | 28 | 18 | 30 | 29 | 30 | **24** 25 | 30 | 20 | **35** 33 |
| | 24 | 26 | 34 | 31 | 38 | 41 | 28 | 29 | 21 | 32 | 30 | 32 | **27** 26 | 35 | **26** 22 | **33** 34 |
| | 25 | 27 | 35 | 32 | 39 | 45 | 29 | 30 | 23 | 33 | 31 | 33 | **26** 27 | 36 | **22** 26 | **36** 35 |
| | 29 | 28 | 36 | 34 | 43 | 47 | 30 | 32 | 24 | 34 | 33 | 34 | 31 | 38 | 27 | **34** 36 |
| | 31 | 30 | 37 | 35 | 45 | 49 | 31 | 33 | **26** 25 | 36 | 34 | **36** 35 | 32 | 40 | **30** 28 | 38 |
| | 34 | 34 | 38 | 36 | 47 | 50 | 34 | 34 | **25** 26 | **38** 37 | 36 | **37** 36 | **34** 33 | 42 | **28** 30 | 39 |
| | 35 | 38 | 41 | 38 | 50 | | 36 | 36 | 28 | **37** 38 | 39 | **35** 37 | **33** 34 | 46 | 31 | 40 |
| | 37 | 39 | 42 | 41 | | | 37 | 37 | 29 | **43** 40 | 40 | 39 | **36** 35 | 49 | **33** 32 | **46** 42 |
| | 39 | 42 | 43 | 42 | | | 40 | 39 | **32** 31 | **40** 41 | **45** 42 | 40 | **37** 36 | | **32** 33 | **44** 43 |
| | 41 | 43 | 45 | 46 | | | 45 | 40 | **31** 32 | **41** 42 | **46** 44 | **46** 44 | **35** 37 | | 38 | **42** 44 |
| | 42 | 45 | 46 | 50 | | | 42 | 41 | 33 | **42** 43 | **44** 45 | **44** 45 | 38 | | 40 | **43** 46 |
| | 43 | 47 | 47 | | | | 44 | 45 | 35 | 45 | **42** 46 | **45** 46 | 39 | | 43 | 48 |
| | 45 | 50 | 48 | | | | 47 | 46 | **41** 40 | 47 | 47 | 49 | 40 | | 44 | 50 |

| | | | | | | |
|---|---|---|---|---|---|---|
| 46 | 50 | **40** 41 | 50 | 50 | 41 | **46** 45 |
| 47 | | 45 | | | **43** 42 | **45** 46 |
| 50 | | 47 | | | **42** 43 | 47 |
| | | 49 | | | 45 | 48 |
| | | 50 | | | **49** 48 | 49 |
| | | | | | **48** 49 | 50 |
