# Supplementary material for: Two-Phase Analysis in Consensus Genetic Mapping
Source: G3 (Bethesda). 2012 May 1;2(5):537–49. doi: 10.1534/g3.112.002428 (PMC3362937; doi:10.1534/g3.112.002428)
Supplement: Supporting Information [file supp_2.5.537_TableS2.pdf]

Table S2 The advantage of the weighted criterion of sum of recombination rates over the voting criterion in the presence of both pure and very noisy data (simulated data of Example 2.2).

**Set #**	**1**	**2**	**3**	**4**	**5**	**6**	**7**	**8**
**se %**	←	**0**		→	←	**10**		→
Ind(cM)	244	182	251	287	552	578	542	462
Cons(cM)	244	182	251	287	567	590	556	468
	1	1	3	4	**4** 1	1	**4** 3	**4** 1
	4	6	5	5	**1** 2	**3** 2	**3** 4	**1** 4
	5	7	6	8	**2** 3	**2** 3	7	6
	8	8	7	10	**3** 4	4	8	8
	9	9	10	11	7	6	9	9
	10	11	15	12	10	**13** 11	11	13
	11	13	20	13	11	**11** 13	13	**19** 17
	12	14	21	20	12	**26** 25	15	**17** 19
	15	16	23	21	13	**25** 26	**17** 17	26
	16	19	25	24	14	**28** 27	**16** 16	27
	19	21	27	27	17	**27** 28	**26** 25	28
	24	24	30	28	21	30	**25** 26	30
	25	26	34	31	23	32	28	32
	26	27	35	32	24	33	29	33
	29	28	36	34	**26** 25	34	30	34
	31	30	37	35	**25** 26	36	31	**36** 35
	34	34	38	36	28	**38** 37	33	**37** 36
	35	38	41	38	29	**37** 38	34	**35** 37
	37	39	42	41	**32** 31	**43** 40	36	39
	39	42	43	42	**31** 32	**40** 41	39	40
	41	43	45	46	33	**41** 42	40	**46** 44
	42	45	46	50	35	**42** 43	**45** 44	**44** 45
	43	47	47		**41** 40	45	**46** 42	**45** 46
	45	50			**40** 41	47	**44** 45	49
	46				45	50	**42** 46	50
	47				47		47	
	50				49			
					50			
