# Supplementary material for: Two-Phase Analysis in Consensus Genetic Mapping
Source: G3 (Bethesda). 2012 May 1;2(5):537–49. doi: 10.1534/g3.112.002428 (PMC3362937; doi:10.1534/g3.112.002428)
Supplement: Supporting Information [file supp_2.5.537_TableS3.pdf]

**Table S3   The lengths of the chromosomes after individual and consensus analysis allowing  to detect the chromosomes with high cost of consensus ordering: data on six BC maize sets**

| Chr # | Phase | set1 | set2 | set3 | set4 | set5 | set6 |
|---|---|---|---|---|---|---|---|
| 1 | *before* | 243.6 | 212.0 | 226.8 | 184.9 | 202.5 | 234.2 |
| | *after* | 245.7 | 212.1 | 226.8 | 184.9 | 203.6 | 234.2 |
| 2 | *before* | 146.5 | 144.8 | 121.2 | 60.5 | 170.3 | 95.5 |
| | *after* | 146.5 | 167.6 | 123.4 | 61.5 | 170.4 | 95.5 |
| | *after-crt* | 146.5 | 144.8 | 123.4 | 61.5 | 170.3 | 95.5 |
| 3 | *before* | 154.1 | 167.8 | 147.9 | 172.8 | 140.3 | 117.0 |
| | *after* | 154.1 | 167.8 | 147.9 | 172.8 | 144.6 | 117.0 |
| 4 | *before* | 100.6 | 158.9 | 142.0 | 144.4 | 123.9 | 105.2 |
| | *after* | 100.6 | 158.7 | 142.0 | 144.4 | 123.9 | 105.2 |
| 5 | *before* | 143.5 | 157.5 | 158.3 | 162.3 | 136.2 | 151.2 |
| | *before-crt* | 143.5 | 157.5 | 135.2 | 162.3 | 136.2 | 151.2 |
| | *after* | 143.5 | 157.5 | 166.4 | 163.5 | 136.2 | 151.2 |
| | *after-crt* | 143.5 | 157.5 | 137.8 | 163.5 | 136.2 | 151.2 |
| 6 | *before* | 104.5 | 139.6 | 53.6 | 120.9 | 125.6 | 83.4 |
| | *after* | 104.5 | 139.8 | 53.6 | 211.8 | 128.9 | 83.4 |
| | *after-crt* | 104.5 | 139.7 | 53.6 | 120.7 | 125.7 | 83.4 |
| 7 | *before* | 111.9 | 139.8 | 191.7 | 111.8 | 124.3 | 92.0 |
| | *before-crt* | 111.9 | 139.8 | 162.8 | 111.8 | 124.3 | 92.0 |
| | *after* | 111.9 | 139.8 | 200.6 | 111.8 | 124.3 | 92.0 |
| | *after-crt* | 111.9 | 139.8 | 167.2 | 111.8 | 124.3 | 92.0 |
| 8 | *before* | 133.4 | 141.8 | 114.5 | 104.6 | 145.7 | 86.7 |
| | *after* | 133.4 | 141.8 | 121.0 | 104.6 | 145.7 | 86.7 |
| 9 | *before* | 132.3 | 119.1 | 136.3 | 139.8 | 97.8 | 46.2 |
| | *after* | 132.3 | 119.1 | 136.3 | 139.8 | 97.8 | 46.2 |
| 10 | *before* | 97.2 | 114.1 | 112.1 | 75.9 | 109.3 | 58.9 |
| | *after* | 97.2 | 114.2 | 115.3 | 75.9 | 111.5 | 58.9 |

Notes: *before* – the lengths (in cM) of the map variants after individual analysis of separate sets but before the consensus analysis; *before-crt* – the same as *before* but after removing some markers of chromosomes 5 and 7 in set #3; *after* - the legths of the map variants after the consensus analysis; *after-crt* – the same as *after* but after removing some markers of set2-Chr2, set3-Chr5, set4-Chr6, and set3-Chr7
