# Supplementary material for: Two-Phase Analysis in Consensus Genetic Mapping
Source: G3 (Bethesda). 2012 May 1;2(5):537–49. doi: 10.1534/g3.112.002428 (PMC3362937; doi:10.1534/g3.112.002428)
Supplement: Supporting Information [file supp_2.5.537_TableS4.pdf]

Table S4 The lengths of the chromosomes after individual and consensus analysis allowing to detect the chromosomes with high cost of consensus ordering: data on 24 RIL maize sets

Chr	b/a	Sets (mapping populations)											
Chr 1		set1	set2	set3	set4	set5	set6	set7	set8	set9	set10	set11	set12
	b	170.5	158.1	198.3	160.8	201.2	175.2	151.8	180.7	166.8	164.1	160.2	157.2
	a	170.6	159.6	199.7	161.5	203.3	175.6	153.4	182.0	169.0	164.9	161.2	159.8
		set14	set15	set16	set18	set19	set20	set21	set22	set23	set24*	set25*	set26
	b	166.8	143.1	157.2	149.8	172.2	188.8	131.0	165.1	113.1	134.4	139.1	179.3
	a	170.1	143.1	157.3	150.3	172.8	189.4	131.0	165.8	113.9	134.8	139.5	179.6
Chr 2		set1	set2	set3	set4	set5	set6	set7	set8	set9	set10	set11	set12
	b	131.5	120.9	161.1	124.5	133.4	150.2	134.0	143.3	123.3	136.2	123.1	148.1
	a	132.0	122.1	161.4	126.1	134.1	150.2	134.4	143.8	124.0	137.1	123.1	148.8
		set14	set15	set16*	set18	set19	set20	set21	set22	set23*	set24*	set25	set26*
	b	112.1	103.3	43.3	116.3	121.8	138.4	64.9	131.2	44.5	39.1	145.8	35.0
	a	112.4	103.3	43.5	116.7	121.8	138.7	64.9	132.0	44.9	39.2	145.8	35.0
Chr 3		set1*	set2	set3	set4	set5	set6	set7	set8	set9	set10	set11	set12
	b	77.1	127.8	137.4	138.5	32.9	97.2	118.0	144.8	120.1	145.6	140.1	143.5
	a	77.1	128.9	138.3	138.7	34.0	97.6	118.0	144.8	120.3	146.9	140.3	143.8
		set14	set15	set16	set18	set19	set20	set21	set22	set23	set24	set25	set26
	b	125.0	-	117.5	107.2	114.8	137.8	135.7	113.1	-	54.4	98.3	80.0
	a	125.1	-	118.4	107.2	115.2	138.1	135.7	113.1	-	54.9	98.3	80.0
Chr 4		set1	set2	set3	set4	set5*	set6	set7	set8	set9	set10	set11	set12
	b	121.3	121.6	132.9	99.0	59.8	65.4	97.4	109.7	111.4	112.0	77.4	72.0
	a	121.6	121.9	133.2	99.6	60.4	65.4	97.9	109.8	112.0	112.0	77.4	72.0
		set14	set15	set16	set18	set19	set20	set21	set22	set23	set24	set25	set26
	b	93.1	49.2	103.4	103.1	113.5	114.9	51.5	125.8	19.5	-	130.7	97.3
	a	93.1	49.2	103.4	103.1	113.5	114.9	51.8	126.1	19.6	-	131.3	100.7
Chr 5		set1	set2	set3	set4	set5	set6	set7	set8	set9	set10	set11	set12
	b	116.	101.	134.6	128.	149.8	147.	122.	143.8	136.4	129.2	119.0	106.5

	2	4		9		4	6					
a	116.9	101.5	137.0	130.1	150.4	148.1	123.2	145.0	136.6	129.5	119.1	107.4
	set14	set15	set16	set18	set19*	set20	set21	set22*	set23	set24	set25	set26
b	113.1	-	104.0	121.6	77.8	104.2	91.2	65.3	-	91.2	96.5	67.5
a	115.8	-	104.9	122.8	78.4	106.3	91.9	66.2	-	91.2	97.1	67.6

Table 4s continued

Chr6		set1	set2	set3	set4	set5	set6	set7*	set8	set9	set10	set11	set12
	b	77.4	92.5	95.2	79.7	96.1	95.0	39.0	101.1	50.1	88.7	83.8	90.5
	a	77.4	93.6	99.0	79.9	96.2	95.0	39.0	101.4	51.0	88.7	83.8	90.6
		set14	set15	set16	set18	set19	set20*	set21	set22	set23	set24	set25	set26
	b	85.9	100.0	82.4	90.6	86.1	38.8	41.8	87.9	15.4	58.2	101.3	107.1
	a	86.2	100.8	82.4	90.8	86.5	38.8	42.4	88.2	15.4	58.2	101.3	107.1
Chr7		set1	set2	set3*	set4*	set5	set6	set7	set8	set9	set10	set11	set12
	b	106.5	103.8	58.8	45.1	65.9	101.7	87.2	108.4	118.8	110.0	105.7	109.3
	a	106.9	103.8	59.2	45.1	65.9	101.7	87.2	112.8	118.8	110.2	105.9	109.6
		set14	set15*	set16	set18	set19	set20	set21	set22	set23	set24	set25	set26
	b	114.6	49.3	107.9	81.9	108.9	68.0	71.4	110.5	49.4	0.3	114.5	105.6
	a	115.3	49.3	108.3	82.2	109.8	68.0	71.7	117.4	49.5	0.3	114.5	107.6
Chr8		set1	set2	set3	set4	set5	set6	set7	set8	set9	set10	set11	set12
	b	107.1	113.3	121.0	91.2	101.3	110.9	69.0	115.7	117.5	116.4	98.4	97.1
	a	107.4	113.6	121.7	92.0	101.6	110.9	69.0	116.1	117.6	116.4	98.8	97.1
		set14	set15	set16	set18	set19	set20	set21	set22	set23	set24	set25	set26
	b	93.5	79.2	105.7	99.1	101.9	96.8	117.5	117.2	62.0	65.4	68.7	116.0
	a	94.1	79.3	106.0	99.1	102.3	96.8	117.5	117.5	62.0	65.4	68.7	116.8
Chr9		set1	set2	set3	set4	set5	set6	set7	set8	set9	set10	set11	set12
	b	89.4	93.2	105.5	97.8	65.9	76.9	91.1	104.5	93.7	105.7	110.0	94.6
	a	89.9	94.1	110.8	98.5	65.9	77.3	91.7	104.5	94.1	106.3	110.9	94.6
		set14	set15	set16*	set18	set19	set20	set21	set22	set23	set24	set25	set26
	b	85.4	80.4	59.7	90.6	92.2	76.5	89.2	94.5	48.4	15.5	106.7	106.5
	a	85.5	81.2	62.6	92.0	92.5	76.6	132.9	94.5	50.2	15.5	106.7	106.9
Chr10		set1	set2	set3	set4	set5	set6	set7	set8	set9	set10	set11	set12
	b	71.6	92.8	98.3	89.8	-	74.2	77.2	81.4	82.9	90.4	91.0	80.1
	a	71.6	93.6	98.3	90.5	-	74.2	77.7	81.4	82.9	90.4	91.0	80.1
		set14*	set15	set16	set18	set19	set20	set21	set22	set23	set24	set25	set26*
	b	53.1	81.8	80.7	73.2	86.8	103.1	75.8	84.5	34.6	58.1	88.6	6.4
	a	53.1	81.8	81.0	73.6	86.8	103.1	75.8	84.8	34.6	58.1	89.0	6.4

Notes: b and a – represent two phases, ***b**efore* and ***a**fter* of the proposed consensus mapping approach. Set-chromosome combinations with high or moderate cost of consensus are marked by frames (especially set21-Chr9). The situation with set21-Chr9 is discussed in more detail in the text. * marks the sets where the considered chromosome is represented by several fragment (linkage groups); for such cases the sum of the fragment lengths (in cM) is presented.
